# Phytochemicals: Essential Oils and Other Extracts for Disease Prevention and Growth Enhancement in Aquaculture: Challenges and Opportunities

**DOI:** 10.3390/ani15182653

**Published:** 2025-09-10

**Authors:** Markos N. Kolygas, Konstantina Bitchava, Cosmas Nathanailides, Foteini Athanassopoulou

**Affiliations:** 1 Department of Aquaculture and Fish Diseases, Faculty of Veterinary Science, University of Thessaly, GR 43100 Karditsa, Greece; kolygasmarkos@gmail.com (M.N.K.); foteini8@gmail.com (F.A.); 2 Laboratory of Applied Hydrobiology, Department of Animal Science, School of Animal Biosciences, Agricultural University of Athens, GR 11855 Athens, Greece; bitchava@aua.gr; 3 Institute of Environment and Sustainable Development (IESD), University Research Center of Ioannina (URCI), GR 45110 Ioannina, Greece; 4 Faculty of Agriculture, University of Ioannina, GR 47100 Arta, Greece

**Keywords:** medicinal aromatic plants, essential oils, plant extracts, fish pathogens

## Abstract

This review explores the potential of essential oils (EOs) as natural feed additives in aquaculture, highlighting their antimicrobial, antioxidant, and immunostimulatory properties, which contribute to disease prevention and improved fish resilience. EOs, derived from aromatic plants, offer a sustainable alternative to synthetic chemicals, promoting benefits such as enhanced growth rates, feed efficiency, immune function, and reduced pathogen susceptibility. However, several challenges must be addressed to fully unravel their potential, including the optimization of dosages, effective delivery methods, and cost-efficiency. Techniques such as microencapsulation are emerging as promising solutions to improve EO stability and controlled release in aquatic feeds, though further research is needed to refine these approaches and evaluate their scalability. Additionally, there is a need for more research into the mechanisms through which EOs influence fish health, the interactions between active compounds, potential synergistic effects of EO mixtures, and their impact on the aquatic environment and microbiome. Addressing these challenges will ensure the effective and sustainable application of EOs in aquaculture, reducing reliance on synthetic chemicals while fostering a more resilient and eco-friendly industry. A key feature of this review is the systematic presentation of detailed, species-specific tables summarizing the current literature on the application of EOs and plant extracts in fish health management.

## Simple Summary

This review examines the potential of essential oils (EOs) as natural additives in aquaculture, emphasizing their antimicrobial, antioxidant, and immune-boosting effects that can improve fish health and resilience. EOs, sourced from plants, offer a sustainable alternative to synthetic chemicals, promoting growth, feed efficiency, and disease resistance. However, challenges such as optimizing dosages, delivery methods, and cost-efficiency remain. Techniques like microencapsulation may improve EO stability and release, but more research is needed to refine these methods and understand how EOs affect fish health and the environment. Overcoming these challenges will enable a more sustainable and eco-friendly aquaculture industry.

## 1. Introduction

The global aquaculture industry has experienced substantial growth, producing approximately 80.0 million tons of various aquatic species in 2024 [1,2], comprising mostly fin fish, mollusks, and crustaceans. This production accounted for over half of the world’s fish consumption for the first time in history [3]. However, infectious diseases pose a significant threat to the profitability of aquaculture, with pond fish aquaculture losing 60% of its production to infectious diseases [4] and the tropical marine shrimp sector experiencing losses of around 40% due to viral diseases [5]. Traditional approaches to address these challenges, such as synthetic growth promoters and antibiotics, have raised concerns regarding food safety and environmental contamination [6].

The overuse of antibiotics in aquaculture has raised concerns about antibiotic resistance and ecological impacts. As a potential solution, researchers are investigating the use of essential oils (EOs). EOs, derived from plants, possess antimicrobial and immunostimulatory properties. Additionally, EOs can enhance fish immune systems, making them more resistant to diseases. This suggests that EOs could be used as natural additives in aquaculture feed to reduce antibiotic usage and improve overall fish health [7]. Given the challenges posed by various bacterial pathogens in aquaculture, the exploration of EOs as a potential alternative is particularly relevant.

Aromatic plants produce aromatic substances, commonly found in EOs, which contain bioactive compounds such as terpenes, terpenoids, aldehydes, ketones, acids, phenols, lactones, ethers, and esters [8]. As a result, there is a growing interest in utilizing aromatic plants to produce functional feeds in aquaculture. These functional feeds aim to promote growth, enhance feed conversion, improve health, and address concerns about food safety and environmental sustainability [9,10].

The use of aromatic plants and their extracts offer a promising eco-friendly strategy to combat fish diseases in aquaculture. Their beneficial properties have been demonstrated through numerous scientific studies, often showing comparable or superior efficacy to synthetic substances like antibiotics [11,12,13,14,15]. Additionally, the use of aromatic plants aligns with societal demands for safe and environmentally friendly food production practices.

Furthermore, aromatic plants and their extracts serve as natural immunostimulants, enhancing the innate immune response of aquatic organisms. Unlike vaccines, which target specific pathogens, immunostimulants improve overall immune function, thereby reducing the susceptibility of fish to various opportunistic pathogens [7,16,17,18]. By promoting stress resistance, growth, appetite, and immune function, aromatic plants contribute to the overall health and well-being of aquatic organisms in aquaculture systems.

The integration of aromatic plants and their extracts into aquaculture practices represents a holistic and sustainable approach to disease management and health promotion. This strategy not only addresses the challenges posed by infectious diseases but also aligns with consumer preferences for safe, eco-friendly, and ethically produced seafood [14,19].

This review assesses the potential of essential oils (EOs) and selected plant extracts as natural feed additives in aquaculture. Based on studies identified in our review, we summarize the species examined, inclusion levels, and reported outcomes. The evidence is organized into sections with species-specific tables that collate the studies identified in our review and their main findings.

Recognizing that taxonomy is sometimes applied unevenly in the relevant literature, we adopted POWO [20] and WoRMS [21] to standardize names, thereby limiting ambiguity and facilitating cross-study synthesis.

## 2. Enhancement Overview of Reported Benefits of Essential Oils (EOs)

The growing body of research on EOs in aquaculture continues to uncover a wide range of benefits for various fish species. EOs such as Cinnamon EO and Origanum EO have been linked to enhanced growth rates, feed conversion efficiency, immune status, disease resistance, and intestinal health in species like *Dicentrarchus labrax* and *Oreochromis niloticus* [22,23,24,25]. El-Sayed et al. (2024) [26], further support the potential benefits of EOs in aquaculture. Their study demonstrated that dietary supplementation with a mixture of botanical compounds and EOs in Nile tilapia significantly improved feed efficiency, antioxidant status, immune parameters, and digestive health.

Thyme EO, particularly when combined with a prebiotic, has shown promising results in *Oncorhynchus mykiss*, significantly enhancing growth performance, digestive enzyme activity, and humoral as well as skin and intestinal immune responses [27]. In *Cyprinus carpio,* the inclusion of *Yucca schidigera* EO improved intestinal antioxidant capacity and immune response [28], while Lavender EO also promoted growth, immune-related gene expression, and reduced stress response [29]. Additionally, *Rosmarinus officinalis* EO has demonstrated the ability to control parasitic infections in *Cyprinus carpio*, particularly against monogenean infections [30].

Moreover, EOs such as Garlic EO and bioactive compounds like Carvacrol and Thymol have been shown to promote skin innate immunity, modulate transcriptional immune responses, and reduce stress and bacterial growth in the mucus of *Sparus aurata* [31]. *Cinnamomum verum* EO has demonstrated its ability to reduce the toxic effects of aflatoxin B1, improving hematological indices, serum biochemistry, and liver histopathology in *Oncorhynchus mykiss* [32]. EOs offer significant potential in promoting growth, immunity, antioxidant capacity, and intestinal health in aquaculture, providing a natural and effective approach to improving fish welfare and performance. These diverse health benefits, particularly improvements in digestion and immune function, contribute to enhanced growth and feed efficiency, paving the way for their use as growth promoters.

In addition to the potential benefits of EOs on growth in aquaculture, their impact on reducing stress is also significant. EOs, such as oregano oil, have been studied for their stress-reducing capabilities in fish, particularly under intensive farming conditions where stress levels are elevated due to high stocking densities and other environmental factors [33]. El-Hawarry et al., in 2018 [33], investigated the combined effects of rearing density and oregano oil supplementation on the growth, behavior, and stress response of Nile tilapia (*Oreochromis niloticus*). The findings demonstrated that oregano oil supplementation positively influenced growth rates and improved behavioral responses, especially under high-density conditions. Additionally, it significantly reduced stress responses in the fish, suggesting that oregano oil can act as an effective stress-reducing agent in aquaculture systems.

The stress-reducing mechanisms of EOs are believed to be linked to their antioxidant properties, which lower oxidative stress, as well as their anti-inflammatory effects, which can help to mitigate stress-induced inflammation, a significant parameter for improving the welfare of fish reared under high-density conditions [34].

Table S1 synthesizes the studies identified in our review, reporting benefits of dietary EOs in aquaculture [22,23,24,25,27,28,29,30,32,35,36,37,38,39,40,41,42,43,44,45,46,47,48,49,50,51,52,53,54,55,56,57,58,59,60,61,62,63,64,65,66,67,68,69,70,71,72,73,74,75,76,77,78,79,80].

## 3. The Role of EOs in Palatability and Growth Enhancement

Fish palatability is a critical factor, influencing the potential benefits of fish feed additives, directly affecting feed intake and overall growth performance [81]. There is evidence suggesting that certain MAPs extracts can actually enhance appetite and improve feed intake in fish when used at appropriate doses. Abdel-Tawwab et al. [82] reported that dietary green tea supplementation improved feed intake and growth performance in Nile tilapia, likely due to enhanced palatability and metabolic stimulation. Similarly, Dawood et al. [83] reviewed several studies reporting that various herbal EOs can enhance fish feed palatability; however, high doses may inhibit feed consumption.

Additionally, garlic stands out as one of the most studied aromatic plants for growth promotion in fish and crustaceans [84]. Studies have shown that incorporating garlic into fish diets enhances consumption, specific growth rates, and weight gain [85]. Similar growth-promoting effects have been observed with other aromatic plants rich in compounds like thymol and carvacrol, such as thyme and oregano. Peppermint has also demonstrated growth-enhancing properties when added to fish diets, with dose-dependent improvements in growth rates [86]. Fenugreek seeds have garnered interest for their growth-stimulating effects in various fish species, showing significant improvements in growth parameters when included in the diet [87]. It is important to note that many studies have also reported beneficial effects of aromatic plant extracts and oils on other aspects of animal health and well-being, though not directly related to growth.

A period of adaptation may be required in some cases; for example, long-term dietary supplementation with lavender oil improved feed utilization and digestive enzyme activities in European seabass without negative effects on feed acceptance [54]. It can be stated that while MAPs can improve palatability and overall performance, a period of adaptation and optimization of dosage is crucial to avoid adverse effects on feed intake.

## 4. The Immunostimulatory Properties of Essential Oils and Applications for Disease Prevention in Aquaculture

Fish possess an immune system, which includes both innate and adaptive components. The innate immune system provides immediate, non-specific defenses against a wide range of pathogens, utilizing physical barriers such as the skin and gills, as well as cellular and humoral factors like phagocytes and complement proteins. The adaptive immune system offers long-lasting and specific protection against pathogens. Immunostimulation seeks to enhance the function of both the innate and adaptive immune systems in fish. By stimulating the production of immune cells, increasing the activity of immune-related genes, and improving the overall immune response, immunostimulation helps fish better defend against diseases [17].

Immunostimulation plays a crucial role in addressing current and future challenges in aquaculture by helping combat emerging diseases and enabling fish populations to develop resistance to pathogens. It can also help mitigate the effects of environmental stressors, such as those affected by climate change, which can compromise fish health. The expected benefits of immunostimulation include improved disease resistance, reducing mortality rates and enhancing overall health. Additionally, immunostimulants can reduce the reliance on antibiotics, thereby mitigating the risk of antibiotic resistance and contributing to the sustainability of aquaculture practices [83,88,89,90]. The mechanism involved inimmunostimulatory effects of EOs include toll-like receptors (TLRs) of immune cells, which play a critical role in recognizing pathogens and triggering immune responses. When TLRs are activated, they stimulate the production of pro-inflammatory cytokines such as IL-1β, which is crucial for initiating and regulating the immune response. The production of IL-1β in response to EOs indicates their potential as immunostimulants, helping fish against infections and diseases [91,92,93,94,95,96,97]. However, it is essential to maintain a balance since excessive IL-1β production could lead to harmful inflammation. Therefore, while EOs can enhance the immune system, their use should be carefully managed. This balance is crucial in ensuring that EOs contribute positively to fish health and well-being without causing detrimental inflammation-related issues [16,98,99,100]. Another benefit of EOs is the functional integrity of gut health in fish. A functionally intact gut is crucial for the immune system of fish, as it helps reduce oxidative stress and enhance nutrient absorption. By reducing pathogenic bacteria and promoting beneficial gut microbiota, EOs contribute to a healthier intestinal environment [16,97,101]. By supporting gut health, EOs can indirectly bolster the immune system, making fish more resilient to diseases and improving their overall well-being.

Medicinal Aromatic Plants (MAPs) offer a promising approach to improving fish health, reducing disease outbreaks, and promoting sustainable aquaculture practices [97,102].

The fight against emerging infectious diseases in aquaculture is of paramount importance for the industry’s profitability and sustainability, as outbreaks continue to inflict significant economic losses globally. These outbreaks often originate from wild hosts in surrounding waters and exploit the compromised immune systems of farmed fish, which can result from stress, confinement, and genetic factors. Over the years, these outbreaks have become a recurrent challenge in aquaculture, typically emerging a few years after the introduction of new species [103].

In response to the limitations and growing concerns surrounding the use of veterinary drugs and synthetic substances in aquaculture [104], aromatic plants and their extracts have garnered increasing attention as potential alternatives. The immunostimulating and health-promoting properties of these plants make them promising candidates for replacing antibiotics and other synthetic medicines. As a result, there has been an exponential growth in research (Table S2.), focusing on the health and immunology of fish concerning the use of aromatic plants [99,105,106,107,108,109,110,111,112,113,114,115,116,117,118,119,120,121,122,123,124,125,126,127,128,129,130,131,132,133,134,135,136,137,138,139,140,141,142,143,144,145,146,147,148,149,150,151,152,153,154,155,156,157,158,159,160,161].

Numerous aromatic plants have been identified for their immunostimulating properties, including garlic, onion, thyme, oregano, rosemary, peppermint, fenugreek, and cumin seeds. These plants contain bioactive compounds such as allicin, thymol, and carvacrol, which have demonstrated significant immunoregulatory effects in fish [99,102,162]. For instance, thymol, a natural compound derived from thyme EO, has gained attention as a potential feed additive in aquaculture due to its antimicrobial, antioxidant, and anti-inflammatory properties. This compound is increasingly studied for its capacity to enhance fish health and reduce dependency on antibiotics [88,102,163]. The benefits of incorporating thymol as a feed additive in aquaculture are substantial. Thymol has been shown to improve fish immune function, thereby reducing disease susceptibility and promoting overall well-being [163,164,165]. Additionally, research indicates that thymol supplementation can enhance growth performance, leading to better growth rates, feed efficiency, and survival in various fish species [102]. Its antimicrobial properties are particularly valuable in controlling bacterial and parasitic infections, which in turn can decrease the need for antibiotic treatments [31,74,166]. Moreover, thymol contributes to improved feed quality by inhibiting spoilage and reducing mycotoxin contamination, ensuring a healthier diet for the fish [73].

Reducing reliance on antibiotics aligns with efforts to minimize the environmental impact of aquaculture by decreasing chemical usage, decreasing parasitic infestations, and mitigating the spread of antibiotic resistance [13,167,168,169].

Garlic and its derivatives have been studied extensively, with research indicating improvements in fish immunity and disease resistance following dietary supplementation [31,170]. Similarly, other compounds such as carvacrol, prevalent in plants like oregano, have shown promise in enhancing immune system function and reducing mortality rates in challenged fish [162,163,164].

Research efforts have also focused on evaluating the immunostimulating effects of aromatic plants in various aquaculture species, including finfish and crustaceans. Studies have investigated the efficacy of different plant extracts and EOs in improving fish health parameters, such as respiratory burst, phagocytic activity, lysozyme activity, and antioxidant enzyme levels [171]. Additionally, the use of aromatic plant extracts has shown potential in controlling bacterial and parasitic infections in aquaculture settings.

While individual plant species exhibit promising immunostimulating effects, the future of disease management in aquaculture could lie in the synergistic combination of multiple plants or phytochemicals [172,173]. However, determining optimal dosages and formulations requires a thorough understanding of the bioactive compounds present in the extracts used. As such, further research is necessary to refine dosages, evaluate efficacy across different aquaculture species, and develop practical applications for integrating aromatic plants into aquafeed formulations [174].

Across the existing literature, there are numerous reports of studies aimed to investigate ways to bolster immunity against specific pathogens. Table S2 presents reported antibacterial and antifungal activity of essential oils, including plant source/part, target pathogen(s), and outcomes. Because extraction methods and chemotypes differ across different studies, we refrain from ranking efficacy. Instead, Table S2 groups in vitro findings by target pathogen and EO/major phenolics (e.g., thymol, carvacrol), to highlight recurring compound classes. We note that MIC values are assay-dependent and do not translate directly to therapeutic doses in vivo; they are best treated as screening indicators.

## 5. Antiparasitic Activity

Infestations of ectoparasites pose significant health risks and economic losses for both saltwater and freshwater aquacultured fish. Among these parasites, monogeneans are particularly problematic as they inhabit the skin, gills, and even the eyes of fish [175]. Traditional treatment methods involve chemotherapy baths, but these can introduce harmful substances into the environment. To address these challenges, aromatic plant extracts and essential oils (EOs) have been assessed for potential anthelmintic effects in in vitro (Table S3) [176,177,178,179,180,181,182,183,184,185,186,187,188,189,190,191,192,193,194,195,196,197,198,199,200,201,202,203,204,205,206,207,208] and in vivo (Table S4) assays [209,210,211,212,213,214,215,216,217,218,219,220,221,222,223]. Garlic, known for its anthelmintic properties, has shown efficacy against monogeneans when administered as a preventive treatment in fish feed. Garlic extract rich in allicin has been effective in reducing infections by *Neobenedenia* sp. when administered orally, as well as in bath therapies [224]. However, it appears to be less effective against juvenile parasites, suggesting its use as a preventive measure rather than a curative treatment.

EOs from other aromatic plants have also shown promise as anthelmintics. Australian tea tree oil has demonstrated dose-dependent reductions in *Gyrodactylus* spp. prevalence when used in baths [225]. Eugenol, found in clove EO, has been effective against monogeneans in tambaqui, though its effects were observed after a week [226]. Additionally, EOs from *Lippia* sp. and clove basil have shown anthelmintic effectiveness in tambaqui.

Rosemary extracts, both ethanolic and aqueous, have shown anthelmintic properties in carp, with the aqueous extract being less toxic to fish [30]. Peppermint EO has demonstrated antiparasitic effects in Nile tilapia, reducing the prevalence of certain monogenean parasites in therapeutic baths. However, the effectiveness and safety of peppermint oil varied depending on the species and concentration used, highlighting the importance of determining optimal doses for each species and pathogens [227].

Overall, natural treatments using extracts and EOs from aromatic plants hold promise as alternative strategies for controlling ectoparasites in aquaculture. Establishing toxicity limits and optimal treatment protocols for each species and pathogen could lead to the adoption of safer and more environmentally friendly treatments, reducing contamination risks for both fish and the environment. Reported in vivo antiparasitic uses span dietary prophylaxis and short therapeutic baths, with effective exposures varying by EO, species, and life stage (e.g., dose-dependent reductions with tea tree oil baths; delayed effects with eugenol; species- and concentration-dependent responses to peppermint; lower-toxicity aqueous rosemary extracts). Given this variability, species-specific range-finding is advisable before routine application, and dietary approaches may be more practical for prevention than repeated baths. We therefore refrain from general dosing recommendations.

Where efficacy and acute toxicity were both reported, effective bath concentrations were typically ≤10–20% of the species-specific 96 h LC_50_ (Table S4), indicating a workable but sometimes narrow safety margin. Given variability across species, life stage, exposure time, and chemotype—and reports of non-target toxicity (e.g., *Daphnia magna*)—species-specific titration and environmental risk assessment are advisable (Table S5) [228,229,230,231,232,233,234,235,236,237,238,239,240,241,242,243,244,245,246,247,248,249,250,251,252,253,254,255,256,257,258,259,260].

## 6. Harnessing Medicinal Aromatic Plants for Sustainable Aquaculture: Challenges and Opportunities

Using MAPs as feed additives presents challenges due to inter-species variability, dose–response effects, and plant-part chemistry. For example, dose optimization is critical for thymol in *Channa argus* [166], tamarind leaf extract in *Oreochromis niloticus* [261], and oregano essential oil in *Ictalurus punctatus* [262]. While MAPs can be beneficial as feed additives, their effectiveness is highly dependent on the appropriate dosage [83]. For example adverse effects may be attributed to strong odors from certain MAPs, which at least initially affected palatability of the feed, leading to reduced feed intake and growth performance [54]. This underscores the need for further research to verify the overall efficiency of MAP dosages in fish feeds [263]. Another challenge associated with the use of EOs in aquaculture is their potential interaction with other feed additives or medications. Certain EOs might exhibit antagonistic or synergistic effects [173] when combined with other compounds in the fish diet. This necessitates thorough research to evaluate the compatibility of EOs with commonly used feed additives and medications to ensure optimal efficacy and avoid unintended consequences [76,264].

Another area of interest involves optimizing the method for incorporation of aromatic plant oils and extracts into fish feed to ensure preservation, controlled release, and effectiveness [265]. Microencapsulation has emerged as a promising method to add EOs to dry feed, preventing interactions with other feed components and preserving active compounds from degradation. This approach transforms the compounds into powder additives, facilitating homogenization in water and incorporation into fish feed [266]. Microencapsulation of aromatic medicinal plants in fish feeds is a promising strategy for boosting the nutritional and therapeutic benefits of aquaculture diets [267]. This technique involves encasing bioactive compounds, such as EOs and plant extracts, in a protective layer to enhance their stability and ensure a controlled release in fish feeds. However, several challenges must be addressed for its successful implementation. The cost of microencapsulation can be high, especially at a commercial scale, which may hinder its widespread use in the aquaculture industry.

Additionally, the encapsulation process can sometimes diminish the bioactivity of these compounds, as the conditions needed for encapsulation might reduce their effectiveness. It has been reported that the effectiveness of MAPs dosages may be influenced by the microencapsulation method used. Spray drying technology based methods have been successfully used for microencapsulation but variations in the release rates of plant EO products between different microcapsule protocols suggest that the delivery and impact of the encapsulated compounds can differ [268], although some studies indicate no significant variation. Liu et al. (2023) [263], found that while both hot and cold spray microencapsulated Origanum oils (MOOs) improved the growth and health of juvenile largemouth black bass, the specific microencapsulation technique had minimal impact on the effectiveness of the dosage. The study showed that the benefits, such as enhanced antioxidant activity and immune responses, were more dependent on the amount of MOO used rather than the method of encapsulation. This suggests that, in this case, the dosage of bioactive compounds is more critical than the microencapsulation technique. Microencapsulation of EOs from medicinal aromatic plants offers significant benefits for protecting the EOs from oxidation, and this is crucial for commercial fish feeds supplemented with EOs of MAPs. Microencapsulation provides protection from degradation by environmental factors like light, temperature, and oxygen, enhancing the stability and longevity of EOs. Furthermore, microencapsulation allows controlled release, ensuring that EOs are delivered effectively to the fish over time, maximizing their beneficial effects while minimizing losses [269]. In turn, this stability improves the handling and storage of EOs, makes the feed more stable by preserving nutrients, and reduces off-flavors, which increases the palatability of the feed for fish [270,271]. Key techniques used in microencapsulation, such as emulsion polymerization, spray drying, and coacervation, are particularly relevant for the fish feed industry because they ensure the effective delivery and sustained efficacy of EOs. Further research is needed to explore microencapsulation protocols with novel wall materials, improve scalability, enhance cost-effectiveness, and conduct more in vivo studies to confirm the benefits of EOs in fish feed [83,272]. This would help in developing more effective and sustainable fish feed formulations.

Another significant challenge is ensuring that the bioactive substances are released consistently within the fish’s digestive system. Despite these obstacles, microencapsulation holds great potential for promoting sustainable aquaculture by allowing the integration of plant-based alternatives into fish feeds, thereby improving fish health and growth while reducing dependence on synthetic additives. Furthermore, there is potential in incorporating oils or extracts into live prey for larvae, aiming to enhance larval development and immune system function, particularly for species with challenging larval phases. Although experiences with white leg shrimp are limited, enriching brine shrimp with garlic extract has shown positive outcomes [273].

Additionally, EOs of aromatic plants are being explored as functional additives in fish feed with high proportions of vegetable ingredients, such as soy meal. High levels of soy meal in marine fish diets can lead to intestinal inflammation [274]. Researchers have investigated the use of aromatic plant extracts and EOs to mitigate these effects. For instance, compounds containing thymol and carvacrol reduced enteritis-related parameters in Japanese sea bass fed diets with partial soy meal replacement. Similarly, a combination of EOs improved protein and fat retention and minimized fecal nitrogen loss in gilthead seabream diets with high vegetable protein content [265].

A potential new application of functional feed additives containing EOs is the development of organic aquaculture. The growing demand for organic seafood has created opportunities for using aromatic plants in aquaculture. Organic certification often requires the avoidance of synthetic additives, making natural alternatives like aromatic plants particularly appealing. By incorporating aromatic plants into organic aquaculture practices, producers can enhance fish health, reduce reliance on synthetic chemicals, and meet the growing demand for sustainable, organic seafood.

While EOs hold significant promise as natural feed additives, several aspects considering their use need further exploration. Research on the long-term effects of EO supplementation on fish physiology and immune function is still limited. Furthermore, more studies are needed to determine the precise mechanisms by which EOs influence fish growth, immune responses, and disease resistance. Future research should also focus on the possible interaction of active compounds of different EOs and any synergistic effects of combining EOs with other natural feed additives, as well as the potential impact of EOs on the aquatic environment and microbiome. While EOs offer promising natural alternatives to synthetic chemicals in aquaculture, it is imperative to conduct thorough environmental risk assessments to ensure their safe and sustainable application. Miura et al. (2021) [275] highlighted the potential toxicity of various EOs to non-target organisms like *Daphnia magna*. Published LC_50_ values for non-target organisms such as *Daphnia magna* vary by EO and test conditions and can overlap with nominal bath concentrations used experimentally for ectoparasites; we flag this as a limitation and a priority for future synthesis and risk assessment. To minimize risks and optimize benefits, studies should focus on determining optimal dosages and delivery methods, such as dietary inclusion or controlled release systems. By understanding the environmental implications and developing targeted application strategies, we can harness the potential of EOs in aquaculture while safeguarding aquatic ecosystems. Addressing these gaps will enable a more comprehensive understanding of how to best utilize EOs in aquaculture, ensuring their effective and sustainable application in the industry.

## 7. Experimental Approaches to the Safety, Efficacy, Genotoxicity, and Developmental Toxicity of EOs

Acute toxicity tests are short-term bioassays and are commonly used to determine the adverse effects of MAPs on aquatic organisms, especially fish. These tests typically measure the LC_50_ (Lethal Concentration 50%), which represents the concentration at which 50% of the test organisms die within a fixed exposure period, often at 24, 48, or 96 h. The aim is to estimate the immediate toxic potential of a compound to help inform safe usage levels, particularly for therapeutic, anesthetic, or antiparasitic applications in aquaculture. Because EOs are complex mixtures of biologically active compounds (like terpenes, alcohols, and phenolics), their toxicity can vary significantly based on the species, life stage, environmental conditions, and exposure time (Table S5).

As already mentioned, EOs are increasingly explored as alternatives to synthetic drugs. However, their natural origin does not inherently guarantee safety. Acute toxicity tests provide critical baseline data for assessing the safety margins of these bioactive agents. Without such evaluations, there is a risk of overdosing, unintended mortality, or sublethal effects such as stress, impaired behavior, or immunosuppression. Therefore, integrating acute toxicity assessments into the early stages of EO application development is vital for ensuring both therapeutic efficacy and ecological compatibility.

Although safety evaluation is essential, comprehensive toxicity testing—particularly through alternatives to traditional animal models—has been limited. Lanzerstorfer et al., in 2021 [276], proposed a strategic approach utilizing both in vitro (cell culture) and alternative in vivo models (such as *Caenorhabditis elegans* and the hen’s egg test) to thoroughly assess the acute, developmental, and reproductive toxicity, as well as the potential mucous membrane irritation, of commonly used EOs.

Table S5 compiles the studies identified in the present review that have reported toxicity tests across different fish species to date.

## 8. Plant and Animal Nomenclature Inconsistencies and Its Importance on Future Pharmacological Evaluation Tests and Aquaculture Use

Plant and animal taxonomy is a fundamental aspect of botanical and ecological research, yet inconsistencies in species nomenclature present significant challenges to scientific communication. This issue arises from multiple factors, including the use of outdated names, typographical errors, synonymous classifications, and the omission of taxonomic authorities. Such discrepancies hinder data accuracy, reproducibility, and interdisciplinary collaboration. Additionally, the lack of standardization in manufacturing validation and experimental methodologies, particularly in low-income countries, further complicates research on plant-derived compounds such as EOs [277]. Addressing these inconsistencies is crucial for advancing research in biodiversity conservation, pharmacognosy, and ecological studies, as well as for ensuring reliable experimental outcomes in toxicology and pharmacological evaluations [278]. Scientific nomenclature serves as the backbone of biological classification, ensuring clarity and uniformity in species identification. However, within the field of botany, plant species are frequently assigned multiple names due to historical revisions, taxonomic reclassification, and regional naming conventions. This creates ambiguity, particularly in academic publications regarding EOs, where inconsistent terminology can obscure research findings and complicate data integration across disciplines. Furthermore, experimental approaches in plant-based research, including toxicological and pharmacological studies, often lack standardized protocols, particularly in resource-limited settings, leading to variations in study outcomes and challenges in cross-study comparisons.

A single species can be described by different researchers, leading to variations in its binomial nomenclature. For example, *Piper aduncum* L. and *Piper aduncum Vell.* ambiguously refer to the same species, yet the latter has been synonymized with *Piper hispidum Sw.* (accepted nomenclature). Without clear citation of the taxonomic authority, misidentification may occur, affecting ecological and pharmacological studies [279].

Taxonomic revisions often result in species being reclassified, rendering previous names obsolete. Many scientific publications, however, continue to use outdated or synonymous names, creating inconsistencies in databases and literature searches. This is evident in medicinal plant research, where traditional and modern nomenclature frequently diverge, leading to confusion in ethnobotanical studies and pharmaceutical applications [280].

Simple typographical errors in species names can propagate throughout the literature, reducing searchability in digital databases and causing misattributions. Omitting the taxonomic authority or incorrectly formatting Latin binomials (e.g., using lowercase subgenus names, or using botanical references (flowers of *Arnica* sp. referred to as *Arnicae anthodium*, which is a botanical reference and not a species)), can further exacerbate identification challenges [281].

The use of vernacular names instead of scientific binomials in publications introduces significant ambiguity [277]. Common names often vary across languages and regions, making them unreliable for precise scientific communication. For instance, the term “mahua” can refer to different species within the *Madhuca* genus, requiring clarification through proper taxonomic citation.

All the above can make future pharmacological evaluation tests and aquaculture use quite challenging. Addressing this need requires not only deeper investigation but also greater taxonomic precision and standardization in reporting. In this context, our study follows up on several previous reviews supporting the immunostimulatory role of MAPs and EOs in aquaculture [84,86,156,158,171,172,173], who emphasize herbal therapies as effective alternatives for enhancing fish immunity and disease resistance. Building on this foundation, our contribution lies in the systematic presentation of detailed, species-specific tables summarizing the current literature on the application of EOs and plant extracts in fish health management. 

## 9. Conclusions

Aromatic plants and their EOs present valuable functional feed additives for aquaculture, offering numerous benefits including enhanced fish health, growth, and disease resistance. These natural compounds align with the industry’s goals of sustainability and environmental responsibility. However, the full potential of EOs can only be realized through addressing key challenges such as optimizing dosages, improving delivery methods, and ensuring cost-effectiveness. Microencapsulation technology holds promise for enhancing the stability and efficacy of EOs in fish feed, but further research is necessary to refine these techniques and explore their scalability. The adoption of EOs in aquaculture can reduce dependency on synthetic chemicals, thereby contributing to a more resilient, eco-friendly, and profitable industry. Continued studies are essential to refine EO applications, optimize their health-promoting properties, and ensure their successful integration into diverse aquaculture systems. In conclusion, embracing aromatic plant-based feed additives presents a pathway not only by improving fish welfare and sustainability but also by addressing the economic and ecological challenges facing modern aquaculture.

## Data Availability

No new data were created or analyzed in this study. Data sharing is not applicable to this article.

## Supplementary Materials

The following supporting information can be downloaded at: https://www.mdpi.com/article/10.3390/ani15182653/s1, Table S1: List of EOs tested in different fish species and their benefits in aquaculture, compiled from published studies identified in the present review. Table S2: Antibacterial and antifungal activities of essential oils and their major compounds against fish pathogens, compiled from published studies identified in the present review. Table S3: In vitro efficacy of different essential oils and their major compounds for different fish species, compiled from published studies identified in the present review. Table S4: In vivo efficacy of different EOs and their major compounds for different fish species, compiled from published studies identified in the present review. Table S5: Acute toxicity tests of different EOs and their major compounds for different fish species, compiled from published studies identified in the present review.
