# Supplementary material for: Phytochemicals: Essential Oils and Other Extracts for Disease Prevention and Growth Enhancement in Aquaculture: Challenges and Opportunities
Source: Animals (Basel). 2025 Sep 10;15(18):2653. doi: 10.3390/ani15182653 (PMC12466357; doi:10.3390/ani15182653)
Supplement: Supplementary file 1 [file animals-15-02653-s001.zip › animals-3796920-supplementary.pdf]

Supplementary Material

Table S1. List of EOstested in different fish species and their benefits in aquaculture.

EO Used	Aquatic Species	Dose	Improved Parameter(s)	Reference
Ajwain	***Chelon ramada*** Risso 1827 (referred to as *Liza ramada*)	0.6 -1g/kg in Feed	Growth Enhancement	[35]
Anise	***Dicentrarchus labrax***Linnaeus 1758	1.5, 2.5, and 3.5 g/kg in Feed	Growth Enhancement	[36]
	Micropterus salmoides Lacepède 1802	0.075% in Feed		[37]
	Oreochromis niloticusLinnaeus 1758	1-2ml/kg in Feed		[38]
Commercial Blend of Star Anise (plus Capsaicin & Saponins)	***Oreochromis niloticus***Linnaeus 1758	0.1-0.4g/kg in Feed	Growth Enhancement	[39]
Artemisia	***Micropterus salmoides*** Lacepède 1802	0.075% in Feed	Growth Enhancement	[37]
Blend of Astragalus/ Forsythia	***Larimichthys crocea*** Richardson 1846	Astragalus 0.1%/Forsythia 0.05 % to 0.15% Feed Ratio	Growth Enhancement	[40]
Blend of Astragalus/ Ginseng (Siberian)	***Larimichthys crocea*** Richardson 1846	Astragalus 0.1%/Siberian Ginseng 0.05 %	Growth Enhancement	[41]
Chaste Tree	***Oncorhynchus mykiss*** Walbaum 1792	50-200mg/kg Feed	Growth Enhancement	[42]
Chia seed	***Labeo rohita*** Hamilton 1822	1% in Feed	Growth Enhancement	[43]
Cinnamon	***Dicentrarchus labrax***Linnaeus 1758	1-2%	Growth,feed utilization, intestinal microbiome	[22]
	Oncorhynchus mykiss Walbaum 1792	1%	Reduced toxic effects of aflatoxin B1, improve liver health indices	[32]
Coriander	***Oreochromis niloticus***Linnaeus 1758	0.5, 10, 15 and 20 g/kg	Growth Enhancement	[44]
Coriander (Vietnamese)	***Oreochromis niloticus***Linnaeus 1758	15-50g/kg in Feed	Growth Enhancement	[45]
Costmary	***Cyprinus carpio***Linnaeus 1758	100-400mg/kg in Feed	Growth Enhancement	[46]
	Oreochromis niloticusLinnaeus 1758	**Diet A.** 1 g/kg yeast (Sc), **Diet B.** 0.1 g/kg costmary EO (Tb), **Diet C.** 1 g/kg yeast + 0.1 g/kg costmary EO (Sc + Tb)		[47]
Dill	***Cyprinus carpio***Linnaeus 1758	1-2% in Feed	No Growth Enhancement	[48]
Fennel	***Salmo labrax*** Pallas 1814	50-400mg/kg in Feed	No Growth Enhancement	[49]
Fenugreek	***Oreochromis niloticus***Linnaeus 1758	0.05, 0.1, 0.15, and 0.2%	Growth Enhancement	[50]
Flaxseed	***Oncorhynchus mykiss*** Walbaum 1792	0.5, 1.0, and 1.5% in Feed	Growth Enhancement	[51]
Laurel	***Oreochromis niloticus***Linnaeus 1758	50mg/Kg &100mg/kg	Growth Enhancement	[52]

	Oreochromis niloticusLinnaeus 1758	1.5g/kg		[53]
Lavender	***Cyprinus carpio***Linnaeus 1758	1.0 & 1.5%	Growth rate, immunity, immune-related gene expression, and stress response	[29]
	Dicentrarchus labraxLinnaeus 1758	2g/kg in Feed	Growth Enhancement	[54]
Lemon (Bitter)	***Oreochromis niloticus*** Linnaeus 1758	0.75⁒, 1⁒	Growth, Blood Parameters, Incrised Immune Response and Oxidative Status	[55]
Lemon Pomace	***Oreochromis niloticus***Linnaeus 1758	1-2% in Feed	Growth Enhancement	[56]
Mooseer	***Cyprinus carpio***Linnaeus 1758	1-2% in Feed	No Growth Enhancement	[48]
Orange	***Colossoma macropomum*** Cuvier 1816	400 -800mg/l	Growth Enhancement	[57]
	Oreochromis niloticusLinnaeus 1758	1⁒, 3⁒	Growth, Blood Parameters, Incrised Immune Response and Oxidative Status	[55]
Oregano	***Dicentrarchus labrax***Linnaeus 1758	0.01%	Growth, digestive enzyme activity, antioxidant defense, immuneresponse	[25]
	Labeo rohita Hamilton 1822	1-1.5% in Feed	Growth Enhancement	[58]
	Oreochromis niloticusLinnaeus 1758	0.5-1.0g/kg in Feed		[59]
	Oreochromis niloticus Linnaeus 1758	0.1-0.2% (a)<br>0.25& 0.5 % (b)	SGR, FCR, immune status and responce, disease resistance and intestinal health	(a)[23]; (b)[24]
Oregano (onites)	***Oncorhynchus mykiss*** Walbaum 1792	0.5 & 3ml/kg in Feed	Growth Enhancement	[60]
Pennyroyal	***Cyprinus carpio***Linnaeus 1758	250 mg/kg in Feed	Growth Enhancement	[61]
Peppermint	***Oreochromis niloticus***Linnaeus 1758	0.6% in Feed	Growth Enhancement	[62]
	Oreochromis niloticusLinnaeus 1758	0.6g/kg in Feed		[63]
Peppermint (in Chitosan nanoparticles)	***Huso baerii*** Brandt 1869(referred to as*Acipenser baerii*)	10g/kg in Feed	Growth Enhancement	[64]
Blend of Peppermint & Clove	***Clarias gariepinus***Burchell 1822	1-2ml/kg in Feed	Growth Enhancement	[65]
Pot Marigold	***Oncorhynchus mykiss*** Walbaum 1792	1.5% in Feed	Growth Enhancement	[66]
Purple Perilla (Shiso)	***Micropterus salmoides*** Lacepède 1802	0.075% in Feed	No Growth Enhancement	[37]
Quebra-faca	***Colossoma macropomum*** Cuvier 1816	0.85ml/kg in Feed	Growth Enhancement	[67]
Rosmary	***Cyprinus carpio***Linnaeus 1758	0.06-0.1%	Control of monogenean infection	[30]
Sage	***Cyprinus carpio***Linnaeus 1758	1-3ml/kg in Feed	No Growth Enhancement	[68]
Savory	***Cyprinus carpio***Linnaeus 1758	2% in Feed	Growth Enhancement	[48]
	Rutilus caspicus Yakovlev 1870	200mg/kg in Feed		[69]
Sea Buckthorn	***Oncorhynchus mykiss*** Walbaum 1792	0.5- 1% in Feed	Growth Enhancement	[70]
Tea Tree	***Oreochromis niloticus***Linnaeus 1758	0.5-2ml/kg in Feed	Growth Enhancement	[71]
Thyme	***Cyprinus carpio***Linnaeus 1758	1-2% in Feed	Growth Enhancement	[72]
	(a) ***Oncorhynchus mykiss*** Walbaum 1792<br>(b) ***Cyprinus carpio***Linnaeus	1%	[1]Reduced the toxic effects of aflatoxin B1, [2] improved growth and immunity status immune-related genes	(a) [73]<br>(b) [74,75]

	1758		[3] intestinal enzymes, digestion,	
Thyme and prebiotic	***Oncorhynchus mykiss*** Walbaum 1792	2% EO 0.2%prebiotic	Growth performance, digestive enzyme activity, humoral immune response, skin and intestinal immune parameters, liver antioxidant capacity	[27]
Blend of Thymol, carvacrol and cinnamaldehyde acid	***Oreochromis niloticus***Linnaeus 1758	3.3g/kg inFeed	Growth Enhancement	[76]
Blend of Thymol, carvacrol, cinnamaldehyde, and monolaurin	***Ctenopharyngodon idella*** Valenciennes 1844<br>***Pelodiscus sinensis*** Wiegmann 1835 (referred to as *Trionyx sinensis*)<br>***Danio rerio***Hamilton 1822	Certain ratio	Growth Enhancement	[77]
Turmeric (Wild)	***Channa punctata*** Bloch 1793	2ml/kg in Feed	Growth Enhancement	[78]
Vernonia Tree	***Oreochromis niloticus***Linnaeus 1758	300-400g/13.89m^3 water	Growth Enhancement	[79]
White Goosefoot	***Oreochromis niloticus***Linnaeus 1758	20-30g/kg in Feed	Growth EnhancementEnhancement	[80]
Yucca	***Cyprinus carpio***Linnaeus 1758	0.04%	Growth, intestinal antioxidant capacityand intestinal immune response	[28]

Table S2. Antibacterial and antifungal activity of different EOs and its major compounds for different fish pathogenic species. Plant nomenclature in this work follows the Plants of the World Online (POWO) database (accessed on April 6th, 2025). If a species name cited in a reference, differs from, or is inconsistent with the updated POWO taxonomy, the referenced name is retained and noted accordingly. **Outcome**: All units are presented exactly as they appear in the original publications. **MIC_{50} (Minimum Inhibitory Concentration 50%)**: The concentration of an antimicrobial agent that inhibits visible growth of 50% of the tested microbial population. **MIC_{90} (Minimum Inhibitory Concentration 90%)**: The concentration that inhibits 90% of the tested microbial strains—used to assess antimicrobial efficacy across a population. **MBC (Minimum Bactericidal Concentration)**: The lowest concentration of an antimicrobial that kills ≥99.9% of the bacterial population. **MFC (Minimum Fungicidal Concentration)**: The lowest concentration of an antifungal agent that kills ≥99.9% of the fungal population. **MBEC (Minimum Biofilm Eradication Concentration)**: The lowest concentration of an antimicrobial required to eradicate a pre-formed microbial biofilm. **MLC (Minimum Lethal Concentration)**: Similar to MBC/MFC; the lowest concentration that kills the test organism, sometimes used more generally or in toxicology contexts. **PTS** (Propyl Propane Thiosulfonate). **PTSO** (Propyl Propane Thiosulfinate Oxide). **PEG400** (Polyethylene Glycol 400). **DMSO** (Dimethyl Sulfoxide). **n/a** (not available information).

Plant Nomenclature	Common name	Plant Part used	Outcome	Pathogen (strain code- and/or aquatic animal, and or Geographical region which was isolated)	Citation
Abies alba Mill.	Fir (Silver)	Needles	MIC_{50} 6.25 MIC_{90} 21.5 (µl/ml)	*Pseudomonas agglomerans*	[105]
			MIC_{50} 12.5 MIC_{90} 25.0 (µl/ml)	*Pseudomonas antarctica*	
			MIC_{50} 6.25 MIC_{90} 12.5 (µl/ml)	*Pseudomonas brassicacearum*	
			MIC_{50} 12.5 MIC_{90} 25 (µl/ml)	*Pseudomonas frederiksbergensis*	
			MIC_{50} 6.25 MIC_{90} 12.5 (µl/ml)	*Pseudomonas koreensis*	
			MIC_{50} 6.25 MIC_{90} 21.5 (µl/ml)	*Pseudomonas lundensis*	
			MIC_{50} 6.25 MIC_{90} 12.5 (µl/ml)	*Pseudomonas mandelii*	
			MIC_{50} 6.25 MIC_{90} 12.5 (µl/ml)	*Pseudomonas proteolytica*	
			MIC_{50} 12.5 MIC_{90} 25.0 (µl/ml)	*Pseudomonas synxantha*	
			MIC_{50} 6.25 MIC_{90} 12.5 (µl/ml)	*Pseudomonas veronii*	

Achillea wilhelmsii K.Koch	Yarrow (achillea)	Aerial parts	MIC 4.0 MFC 8.0 (µg/ml)	*Saprolegnia parasitica*	[106]
Achyrocline saturejoides (Lam.) (referred to as *Achyrocline satureioides*)	Marcela	n/a	MIC >6.4 MBC >6.4 (mg/ml)	*Aeromonas hydrophila*	[107]
			MIC >6.4 MBC >6.4 (mg/ml)	*Citrobacter freundii*	
Alhagi maurorum Medik.	Camelthorn	Aerial parts	MIC >1000 MLC >1000 (µl/ml)	*Lactococcus garviae*	[108]
Allium cepa L.	Onion	Bulb	MIC 2.5 (mg/ml)	*Aeromonas hydrophila*	[109]
			MBC PTS 625.0; MBC PTSO 78.125 (µg/ml)	*Photobacterium damselae subsp. damselae*	[110]
			MBC PTS 625.0; MBC PTSO 156.25 (µg/ml)	*Photobacterium damselae subsp. piscicida*	
			MBC PTS 312.5; MBC PTSO 39.06 (µg/ml)	*Pseudomonas anguilliseptica*	
			MIC 2.5 (mg/ml)	*Pseudomonas fluorescens*	[109]
			MBC PTS 2500.0; MBC PTSO 1250.0 (µg/ml)	*Tenacibaculum maritimum*	[110]
Allium sativum L.	Garlic	stalk	(Peg400) MIC 5415.0 MBC 10830.0; (DMSO) MIC 5415.0 MBC 10830.0 (% v/v)	*Aeromonas hydrophila*	[111]
			(Peg400) MIC 10830.0 MBC 21660.0; (DMSO) MIC 10830.0 MBC 21660.0 (% v/v)	*Aeromonas jandaei*	
		Edible parts	MIC 0.5 MBC 1.0 (µl/ml)	*Lactococcus garviae*	[112]
		Bulb	MIC 0.6 (mg/ml)	*Aeromonas hydrophila*	[109]
			MIC 0.6 (mg/ml)	*Pseudomonas fluorescens*	
			MIC 2.5 (mg/ml)	*Edwardsiella tarda*	
Allium tuberosum Rottler ex Spreng.	Chinese chive	Leaves	MIC 40.0 (µg/ml)	*Flavobacterium columnare (ATCC49512)*	[113]
			MIC 40.0 (µg/ml)	*Flavobacterium columnare (FC1)*	

			MIC 20.0 (µg/ml)	*Flavobacterium columnare (FC2)*	
			MIC 80.0 (µg/ml)	*Flavobacterium columnare (FC2)*	
Amyris balsamifera L.	Amyris	Wood bark	MIC 12.5 (µl/ml)	*Aeromonas* spp.	[114]
			MIC 12.5 (µl/ml)	*Aeromonas salmonicida*	
			MIC 3.12 (µl/ml)	*Enterococcus faecium*	
			MIC 12.5 (µl/ml)	*Pseudomonas fluorescens*	
			MIC 6.25 (µl/ml)	*Yersinia* spp.	
			MIC 12.5 (µl/ml)	*Yersinia ruckeri*	
Anethum graveolens L.	Indian Dill	Seeds	MIC 62.4 MBC 125.0 (µg/ml)	*Lactococcus garviae*	[115]
Angelica sinensis(Oliv.) Diels	Angelica root	Whole plant	MIC 12.5 MBC 25.0 (µl/ml)	*Pseudomonas fluorescens*	[116]
Aniba parviflora (Meisn.) Mez	Pau-rosa	Leaves	MIC 0.8 MBC 1.6 (mg/ml)	*Aeromonas hydrophila*	[107]
			MIC 3.2 MBC 6.4 (mg/ml)	*Citrobacter freundii*	
Aniba rosodoraDucke (referred to as *Aniba rosaeodora*)	Rosewood Pau-rosa	Leaves	MIC 3.2 MBC 3.2 (mg/ml)	*Aeromonas hydrophila*	[107]
			MIC 3.2 MBC 3.2 (mg/ml)	*Citrobacter freundii*	
Apium graveolens L.	Celery	seed	MIC 125.0 MBC 125.0 (µl/ml)	*Pseudomonas fluorescence*	[117]
			MIC 3.9 MBC 3.9 (µl/ml)	*Vibrio alginolyticus*	
**Arnica sp. (**referred to as *Arnicae anthodium* which is a botanical reference and not a species)	Arnica	Dried Plant parts	MIC 0.1 MBC 0.1 (µg/ml)	*Pseudomonas aeruginosa*	[118]
Arnica Montana L.	Arnica	Dried Plant parts	MIC 4.69 (µg/ml)	*Candida albicans*	[118]
Artemisia annua L.	Wormwood (Sweet)	Aerial parts	MIC 3.6 MFC 7.2 (ppm)	*Saprolegnia* sp.	[119]

			MIC 15.6<br>MFC 31.2<br>(ppm)		*Fusarium solani*	
			MIC 6.2<br>MFC 12.4<br>(ppm)		*Aspergillus flavus*	
			MIC 12.4<br>MBC 24.8<br>(µg/ml)		*Streptococcus iniae (LMG14520)*	
			MIC 1.6<br>MBC 3.2<br>(µg/ml)		*Yersinia ruckeri (KC291153)*	
			MIC 3.2<br>MBC >6.4<br>(µg/ml)		*Aeromonas hydrophila (LMG3770)*	
			MIC 6.4<br>MBC 12.8<br>(µg/ml)		*Lactococcus garviae*	
Artemisia vulgaris L.	Mugwort	Leaves	(Peg400)<br>MIC 18610.0<br>MBC 18610.0	(DMSO)<br>MIC 37220.0<br>MBC 37220.0	*Aeromonas hydrophila*	[111]
			(% v/v)			
			(Peg400)<br>MIC 18610.0<br>MBC 18610.0	(DMSO)<br>MIC 37220.0<br>MBC 37220.0	*Aeromonas jandaei*	
			(% v/v)			
Bacopa scoparioides(Cham. & Schltdl.) Scatigna (referred to as *Conobea scoparioides*)	Pataqueira	Leaves	MIC 0.2<br>MBC 0.2<br>(mg/ml)		*Aeromonas hydrophila*	[107]
			MIC 3.2<br>MBC 3.2<br>(mg/ml)		*Citrobacter freundii*	
Bergera koenigii L. (referred to as *Murraya koenigii*	Curry leaf	Several plant parts	MIC 0.39<br>(µg/ml)		*Streptococcus agalactiae*	[120]
Boswellia sacra Flück. (referred to as *Boswellia carterii*)	Frankincense (carterii)	Trunk exuded resin	MIC 12.5<br>(µl/ml)		*Aeromonas* spp.	[114]
			MIC 12.5<br>(µl/ml)		*Aeromonas salmonicida*	
			MIC 3.12<br>(µl/ml)		*Enterococcus faecium*	
			MIC 25.0<br>(µl/ml)		*Pseudomonas fluorescens*	
			MIC 6.25<br>(µl/ml)		*Yersinia* spp.	
			MIC 12.5<br>(µl/ml)		*Yersinia ruckeri*	
			(Peg400)<br>MIC 16952.0<br>MBC 16952.0	(DMSO)<br>MIC 33904.0<br>MBC 33904.0	*Aeromonas hydrophila*	[111]

			(% v/v)			
			(Peg400) MIC 16952.0 MBC 16952.0 (% v/v)	(DMSO) MIC 8476.0 MBC 16952.0	*Aeromonas jandaei*	
Calotropis gigantean (L.) W.T.Aiton	Crown flower	Fresh leaves	MIC 10.0 (mg/ml)		*Aeromonas hydrophila*	[109]
			MIC 10.0 (mg/ml)		*Pseudomonas fluorescens*	
			MIC 0.6 (mg/ml)		*Edwardsiella tarda*	
Camphora officinarumBoerh. Ex Fabr. (referred to as *Cinnamomum camphora*)	Camphor (Standard)	Wood bark	MIC 3.12 (µl/ml)		*Aeromonas* spp.	[114]
			MIC 3.12 (µl/ml)		*Aeromonas salmonicida*	
			MIC 1.56 (µl/ml)		*Enterococcus faecium*	
			MIC 25.0 (µl/ml)		*Pseudomonas fluorescens*	
			MIC 25.0 (µl/ml)		*Yersinia* spp.	
			MIC 25.0 (µl/ml)		*Yersinia ruckeri*	
		Leaf	MIC 625.0 (µg/ml)		*Pseudomonas aeruginosa*	[121]
			MIC 625.0 (µg/ml)		*Serratia marcescens*	
			MIC 312.0 (µg/ml)		*Candida albicans*	
		Branch	MIC 625.0 (µg/ml)		*Pseudomonas aeruginosa*	
			MIC 625.0 (µg/ml)		*Serratia marcescens*	
			MIC 312.0 (µg/ml)		*Candida albicans*	
		Wood	MIC 625.0 (µg/ml)		*Pseudomonas aeruginosa*	
			MIC 39.1 (µg/ml)		*Serratia marcescens*	
			MIC 312.0 (µg/ml)		*Candida albicans*	
		Root	MIC 312.0 (µg/ml)		*Pseudomonas aeruginosa*	
			MIC 625.0 (µg/ml)		*Serratia marcescens*	
			MIC 312.0 (µg/ml)		*Candida albicans*	
		Leaf/branch	MIC 625.0 (µg/ml)		*Pseudomonas aeruginosa*	

			MIC 625.0 (μg/ml)	*Serratia marcescens*	
			MIC 312.0 (μg/ml)	*Candida albicans*	
		Leaf/ branch/ wood	MIC 625.0 (μg/ml)	*Pseudomonas aeruginosa*	
			MIC 625.0 (μg/ml)	*Serratia marcescens*	
			MIC 312.0 (μg/ml)	*Candida albicans*	
Camphora officinarum* var. *officinarum(referred to as *Cinnamomum camphora* var. *Linalolifera*)	Camphor (linalolifera)	Trunks	MIC 3.12 (μl/ml)	*Aeromonas* spp.	[114]
			MIC 3.12 (μl/ml)	*Aeromonas salmonicida*	
			MIC 1.56 (μl/ml)	*Enterococcus faecium*	
			MIC 25.0 (μl/ml)	*Pseudomonas fluorescens*	
			MIC 25.0 (μl/ml)	*Yersinia* spp.	
			MIC 12.5 (μl/ml)	*Yersinia ruckeri*	
Cananga odorata(Lam.) Hook.f. & Thomson	Ylang Ylang	Flower	(Peg400) MIC 36600.0 MBC 36600.0; (DMSO) MIC 36600.0 MBC 36600.0 (% v/v)	*Aeromonas hydrophila*	[111]
			(Peg400) MIC 18300.0 MBC 18300.0; (DMSO) MIC 18300.0 MBC 18300.0 (% v/v)	*Aeromonas jandaei*	
Canarium luzonicum (Blume) A.Gray	Elemi	Trunk exuded resin	MIC 12.5 (μl/ml)	*Aeromonas* spp.	[114]
			MIC 12.5 (μl/ml)	*Aeromonas salmonicida*	
			MIC 6.25 (μl/ml)	*Enterococcus faecium*	
			MIC 25.0 (μl/ml)	*Pseudomonas fluorescens*	
			MIC 25.0 (μl/ml)	*Yersinia* spp.	
			MIC 12.5 (μl/ml)	*Yersinia ruckeri*	
Carum carviL.	Caraway	Seeds	MIC 0.938 MBC 3.750 (mg/ml)	*Vibrio alginolyticus*	[122]
			MIC 0.234 MBC 1.875 (mg/ml)	*Vibrio parahaemolyticus*	

			MIC 0.059<br>MFC 0.469<br>(mg/ml)	*Candida albicans*	
			MIC 0.022<br>MBC 11.562<br>(mg/ml)	*Aeromonas hydrophila*	[123]
			MIC 0.045<br>MBC 5.781<br>(mg/ml)	*Listonella anguillarum*	
			MIC 0.022<br>MBC 11.562<br>(mg/ml)	*Vibrio alginolyticus (ATCC 33787)*	
			MIC 0.045<br>MBC 2.890<br>(mg/ml)	*Vibrio alginolyticus (ATCC 17749)*	
			MIC 0.022<br>MBC 5.781<br>(mg/ml)	*Vibrio harveyi (ATCC 18293)*	
			MIC 0.022<br>MBC 11.562<br>(mg/ml)	*Vibrio parahaemolyticus (ATCC 17802)*	
			MIC 0.022<br>MBC 2.890<br>(mg/ml)	*Vibrio parahaemolyticus (ATCC 43996)*	
		Fruits	MIC_{50} 25.0<br>MIC_{90} 50.0<br>(µl/ml)	*Pseudomonas agglomerans*	[105]
			MIC_{50} 50.0<br>MIC_{90} 100.0<br>(µl/ml)	*Pseudomonas antarctica*	
			MIC_{50} 50.0<br>MIC_{90} 100.0<br>(µl/ml)	*Pseudomonas brassicacearum*	
			MIC_{50} 12.5<br>MIC_{90} 25.0<br>(µl/ml)	*Pseudomonas frederiksbergensis*	
			MIC_{50} 12.5<br>MIC_{90} 25.0<br>(µl/ml)	*Pseudomonas koreensis*	
			MIC_{50} 25.0<br>MIC_{90} 50.0<br>(µl/ml)	*Pseudomonas lundensis*	
			MIC_{50} 25.0<br>MIC_{90} 50.0<br>(µl/ml)	*Pseudomonas mandelii*	
			MIC_{50} 12.5<br>MIC_{90} 25.0<br>(µl/ml)	*Pseudomonas proteolytica*	

			MIC_{50} 25.0 MIC_{90} 50.0 (µl/ml)	*Pseudomonas synxantha*	
			MIC_{50} 25.0 MIC_{90} 50.0 (µl/ml)	*Pseudomonas veronii*	
Cedrus atlantica (Endl.) Manetti ex Carrière	Cedarwood (Atlas)	Wood bark	MIC_{50}: 15.36 MIC_{90}: 23.38 (µl/ml)	*Pseudomonas aeruginosa*	[124]
			MIC 5.0 MBC 5.0 (µl/ml)	*Pseudomonas aeruginosa*	[125]
			MIC_{50} 21.18 MIC_{90} 35.16 (µl/ml)	*Pseudomonas fluorescence*	[124]
			MIC_{50} 21.43 MIC_{90} 32.36 (µl/ml)	*Serratia marcescens*	
			MIC_{50} 17.32 MIC_{90} 22.62 (µl/ml)	*Candida albicans*	
		Cones	MIC 10.0 (µg/ml)	*Proteus vulgaris*	[126]
		Wood bark	MIC 5.0 MFC 5.0 (µg/ml)	*Candida albicans*	[125]
Chamaemelum nobile (L.) All.	Common Chamomile (Roman)	Flower leaves	MIC 5.0 MBC 5.0 (µl/ml)	*Pseudomonas aeruginosa*	[124]
			MIC 5.0 MFC 5.0 (µg/ml)	*Candida albicans*	
Chamaemelum nobile(L.) All. (referred to as *Anthemis nobilis*)		Leaves	MIC 6.4 MBC 6.4 (mg/ml)	*Aeromonas hydrophila*	[107]
			MIC >6.4 MBC >6.4 (mg/ml)	*Citrobacter freundii*	
Chrysopogon zizanioides(L.) Roberty (referred to as *Vetiveria zizanioides*)	Vetiver	Fibrous roots	MIC 12.5 (µl/ml)	*Aeromonas* spp.	[114]
			MIC 12.5 (µl/ml)	*Aeromonas salmonicida*	
			MIC 6.25 (µl/ml)	*Enterococcus faecium*	
			MIC 25.0 (µl/ml)	*Pseudomonas fluorescens*	
			MIC 25.0 (µl/ml)	*Yersinia* spp.	
			MIC 25.0 (µl/ml)	*Yersinia ruckeri*	

***Cinnamomum verum*J. Presl**	Cinnamon (Common)	Wood bark	(Peg400) MIC 662.5 MBC 1325.0	(DMSO) MIC 1325.0 MBC 2650.0	*Aeromonas hydrophila*	[111]
			(% v/v)			
			(Peg400) MIC 2650.0 MBC 2650.0	(DMSO) MIC 2650.0 MBC 2650.0	*Aeromonas jandaei*	
			(% v/v)			
			MIC 120.0 MBC 120.0 (µg/ml)		*Lactococcus garviae*	[127]
***Cinnamomum verum*J. Presl(referred to as *Cinnamomum zeylanicum*)**	Cinnamon (Ceylon)	Wood bark	MIC 0.5 MBC 0.5 (µl/ml)		*Lactococcus garviae*	[112]
			MIC_{50} 3.125 MIC_{90} 6.25 (µl/ml)		*Pseudomonas agglomerans*	[105]
			MIC_{50} 6.25 MIC_{90} 12.5 (µl/ml)		*Pseudomonas antarctica*	
			MIC_{50} 3.125 MIC_{90} 6.25 (µl/ml)		*Pseudomonas brassicacearum*	
			MIC_{50} 6.25 MIC_{90} 12.5 (µl/ml)		*Pseudomonas frederiksbergensis*	
			MIC_{50} 12.5 MIC_{90} 25.0 (µl/ml)		*Pseudomonas koreensis*	
			MIC_{50} 3.125 MIC_{90} 6.25 (µl/ml)		*Pseudomonas lundensis*	
			MIC_{50} 6.25 MIC_{90} 12.5 (µl/ml)		*Pseudomonas mandelii*	
			MIC_{50} 3.125 MIC_{90} 6.25 (µl/ml)		*Pseudomonas proteolytica*	
			MIC_{50} 6.25 MIC_{90} 12.5 (µl/ml)		*Pseudomonas synxantha*	
			MIC_{50} 12.5 MIC_{90} 25.0 (µl/ml)		*Pseudomonas veronii*	
			MIC 0.003 MBC 0.007 (% v/v)		*Photobacterium damselae (FP4101)*	[128]
			MIC 0.003 MBC 0.015 (% v/v)		*Edwardsiella tarda (FP5060)*	

			MIC 0.003 MBC 0.015 (% v/v)	*Edwardsiella tarda (ED47)*	
			MIC 0.001 MBC 0.003 (% v/v)	*Edwardsiella tarda (Yoshida)*	
			MIC 0.001 MBC 0.007 (% v/v)	*Edwardsiella tarda (ED45)*	
			MIC 0.015 MBC 0.031 (% v/v)	*Lactococcus garviae (FP5245)*	
			MIC 0.031 MBC 0.062 (% v/v)	*Streptococcus iniae (FP3287)*	
			MIC 0.015 MBC 0.031 (% v/v)	*Streptococcus iniae (S186)*	
			MIC 0.015 MBC 0.062 (% v/v)	*Streptococcus iniae (S530)*	
			MIC 0.015 MBC 0.062 (% v/v)	*Streptococcus iniae (S131)*	
			MIC 0.07 MBC 0.031 (% v/v)	*Streptococcus parauberis (S124)*	
			MIC 0.007 MBC 0.015 (% v/v)	*Streptococcus parauberis (S527)*	
			MIC 0.007 MBC 0.015 (% v/v)	*Streptococcus parauberis (1466)*	
Citrus x aurantiifolia (Christm.) Swingle	Lime	Fruit peels	MIC 0.031 MBC 0.125 (%v/v)	*Streptococcus parauberis (S124)*	[129]
			MIC 0.125 MBC 1 (%v/v)	*Vibrio harveyi*	
			MIC 0.125 MBC 0.5 (%v/v)	*Vibrio ichthyoenteri*	
			MIC 0.062 MBC 0.125 (%v/v)	*Photobacterium damselae*	
			MIC 0.062 MBC 0.125 (%v/v)	*Photobacterium damselae*	
			MIC 0.25 MBC 1 (%v/v)	*Edwardsiella tarda (ED47)*	
			MIC 0.125 MBC 1 (%v/v)	*Lactococcus garviae*	
			MIC 0.125 MBC 1 (%v/v)	*Streptococcus iniae*	

Citrus x aurantium* f. *deliciosa (Ten.) M.Hiroe (referred to as Citrus *x clementina*)	Clementine	Fruit peels	Sensitive	*Vibrio harveyi*	[130]
			Sensitive	*Listonella anguillarum*	
Citrus x aurantium* f. *deliciosa (Ten.) M.Hiroe(referred to as *Citrus x deliciosa*)	Mandarin (Yellow)	Fruit peels	Sensitive	*Vibrio harveyi*	[130]
			Sensitive	*Listonella anguillarum* (referred to as *Vibrio anguillarum*)	
			MIC 2.5 (mg/ml)	*Pseudomonas fluorescens*	[109]
			MIC 2.5 (mg/ml)	*Edwardsiella tarda*	
Citrus x aurantium L.	Orange (Bitter) Pummelo	Fruit peels	Sensitive	*Vibrio harveyi*	[130]
			Sensitive	*Listonella anguillarum* (referred to as *Vibrio anguillarum*)	
			MIC 25.0 (μl/ml)	*Aeromonas* spp.	[114]
			MIC 12.5 (μl/ml)	*Aeromonas salmonicida*	
			MIC 6.25 (μl/ml)	*Enterococcus faecium*	
			MIC 25.0 (μl/ml)	*Pseudomonas fluorescens*	
			MIC 12.5 (μl/ml)	*Yersinia* spp.	
			MIC 12.5 (μl/ml)	*Yersinia ruckeri*	
		Flower Leaves	MIC 5.0 MBC 5.0 (μl/ml)	*Pseudomonas aeruginosa*	[125]
		n/a	MIC_{50} 98.64 MIC_{90} 101.27 (μl/ml)	*Pseudomonas aeruginosa*	[131]
			MIC_{50} 0.75 MIC_{90} 1.13 (μl/ml)	*Candida albicans*	
		Flower Leaves	MIC 5.0 MFC 5.0 (μg/ml)	*Candida albicans*	[125]
		Leaves Branches Fruits	(Peg400) MIC 35560.0 MBC 35560.0; (DMSO) MIC >35560.0 MBC >35560.0 (% v/v)	*Aeromonas hydrophila*	[111]
			(Peg400) MIC 35560.0 MBC 35560.0; (DMSO) MIC >35560.0 MBC >35560.0	*Aeromonas jandaei*	

			(% v/v)		
		Pericarp	MIC_{50} 25.0 MIC_{90} 50.0 (μl/ml)	*Pseudomonas agglomerans*	[105]
			MIC_{50} 50.0 MIC_{90} 100.0 (μl/ml)	*Pseudomonas antarctica*	
			MIC_{50} 25.0 MIC_{90} 50.0 (μl/ml)	*Pseudomonas brassicacearum*	
			MIC_{50} 50.0 MIC_{90} 100.0 (μl/ml)	*Pseudomonas frederiksbergensis*	
			MIC_{50} 12.5 MIC_{90} 25.0 (μl/ml)	*Pseudomonas koreensis*	
			MIC_{50} 50.0 MIC_{90} 100.0 (μl/ml)	*Pseudomonas lundensis*	
			MIC_{50} 50.0 MIC_{90} 100.0 (μl/ml)	*Pseudomonas mandelii*	
			MIC_{50} 50.0 MIC_{90} 100.0 (μl/ml)	*Pseudomonas proteolytica*	
			MIC_{50} 50.0 MIC_{90} 100.0 (μl/ml)	*Pseudomonas synxantha*	
			MIC_{50} 50.0 MIC_{90} 100.0 (μl/ml)	*Pseudomonas veronii*	
Citrus x aurantium* f. *aurantium(referred to as *Citrus x sinensis*)	Orange	Fruit peels	Sensitive	*Vibrio harveyi*	[130]
			Sensitive	*Listonella anguillarum* (referred to as *Vibrio anguillarum*)	
			MIC 125.0 MBC 250.0 (μl/ml)	*Vibrio vulnificus*	[132]
			MIC >1000.0 MBC >1000.0 (μl/ml)	*Aeromonas hydrophila*	
			MIC >1000.0 MBC >1000.0 (μl/ml)	*Vibrio parahaemolyticus*	
		Pericarp	MIC_{50} 25.0 MIC_{90} 50.0 (μl/ml)	*Pseudomonas agglomerans*	[105]
			MIC_{50} 50.0 MIC_{90} 100.0 (μl/ml)	*Pseudomonas antarctica*	

			MIC_{50} 25.0<br>MIC_{90} 50.0<br>(µl/ml)	*Pseudomonas brassicacearum*	
			MIC_{50} 50.0<br>MIC_{90} 100.0<br>(µl/ml)	*Pseudomonas frederiksbergensis*	
			MIC_{50} 25.0<br>MIC_{90} 50.0<br>(µl/ml)	*Pseudomonas koreensis*	
			MIC_{50} 50.0<br>MIC_{90} 100.0<br>(µl/ml)	*Pseudomonas lundensis*	
			MIC_{50} 25.0<br>MIC_{90} 50.0<br>(µl/ml)	*Pseudomonas mandelii*	
			MIC_{50} 50.0<br>MIC_{90} 100.0<br>(µl/ml)	*Pseudomonas proteolytica*	
			MIC_{50} 50.0<br>MIC_{90} 100.0<br>(µl/ml)	*Pseudomonas synxantha*	
			MIC_{50} 50.0<br>MIC_{90} 100.0<br>(µl/ml)	*Pseudomonas veronii*	
Citrus x bergamia(Risso) *Risso & Poit.* (referred to as *Citrus bergamia*)	Bergamot	Fruit peels	Sensitive	*Vibrio harveyi*	[130]
			Sensitive	*Listonella anguillarum*	
			(Peg400) MIC 8740.0 MBC 8740.0<br>(DMSO) MIC 17480.0 MBC 17480.0<br>(% v/v)	*Aeromonas hydrophila*	[111]
			(Peg400) MIC 17480.0 MBC 17480.0<br>(DMSO) MIC 8740.0 MBC 8740.0<br>(% v/v)	*Aeromonas jandaei*	
Citrus x limon (L.) Osbeck	Eureka Lemon	Fruit peels	Reduced mortalities	*Edwardsiella tarda*	[133]
			Sensitive	*Vibrio harveyi*	[130]
			Sensitive	*Listonella anguillarum* (referred to as *Vibrio anguillarum*)	
			MIC 6.25<br>(µl/ml)	Aeromonas spp.	[114]
			MIC 12.5<br>(µl/ml)	*Aeromonas salmonicida*	
			MIC 6.25<br>(µl/ml)	*Enterococcus faecium*	
		n/a	MIC_{50} 5.56<br>MIC_{90} 7.23<br>(µl/ml)	*Pseudomonas aeruginosa*	[131]

			MIC_{50} 1.21 MIC_{90} 3.18 (µl/ml)	*Candida albicans*	
		Fruit peels	MIC 25.0 (µl/ml)	*Pseudomonas fluorescens*	[114]
			MIC 12.5 (µl/ml)	*Yersinia* spp.	
			MIC 12.5 (µl/ml)	*Yersinia ruckeri*	
			Sensitive	*Aeromonas hydrophila*	[134]
Coriandrum sativum L.	Coriander	Seeds	MIC 0.234 MBC 1.875 (mg/ml)	*Vibrio alginolyticus*	[122]
			MIC 1.875 MBC 7.500 (mg/ml)	*Vibrio parahaemolyticus*	
		n/a	MIC_{50} 11.56 MIC_{90} 13.35 (µl/ml)	*Pseudomonas aeruginosa*	[131]
			MIC_{50} 5.56 MIC_{90} 7.23 (µl/ml)	*Candida albicans*	
		Seeds	MIC 0.469 MFC 1.875 (mg/ml)	*Candida albicans*	[122]
		Dried fruit	MIC_{50} 12.5 MIC_{90} 25.0 (µl/ml)	*Pseudomonas agglomerans*	[105]
			MIC_{50} 12.5 MIC_{90} 50.0 (µl/ml)	*Pseudomonas antarctica*	
			MIC_{50} 25.0 MIC_{90} 50.0 (µl/ml)	*Pseudomonas brassicacearum*	
			MIC_{50} 12.5 MIC_{90} 25.0 (µl/ml)	*Pseudomonas frederiksbergensis*	
			MIC_{50} 25.0 MIC_{90} 50.0 (µl/ml)	*Pseudomonas koreensis*	
			MIC_{50} 6.25 MIC_{90} 12.5 (µl/ml)	*Pseudomonas lundensis*	
			MIC_{50} 6.25 MIC_{90} 12.5 (µl/ml)	*Pseudomonas mandelii*	
			MIC_{50} 12.5 MIC_{90} 25.0 (µl/ml)	*Pseudomonas proteolytica*	

			MIC_{50} 12.5<br>MIC_{90} 25.0<br>(μl/ml)		*Pseudomonas synxantha*	
			MIC_{50} 12.5<br>MIC_{90} 25.0<br>(μl/ml)		*Pseudomonas veronii*	
Corymbia citriodora (Hook.) K.D.Hill & L.A.S.Johnson	Gum (Lemon scented)	Leaves	(Peg400)<br>MIC 4315.0<br>MBC 4315.0	(DMSO)<br>MIC 17260.0<br>MBC 17260.0	*Aeromonas hydrophila*	[111]
			(% v/v)			
			(Peg400)<br>MIC 17260.0<br>MBC 17260.0	(DMSO)<br>MIC 34520.0<br>MBC 34520.0	*Aeromonas jandaei*	
			(% v/v)			
Cuminum cyminum L.	Cumin	Aerial parts	MIC 0.5 MFC 0.5<br>(μg/ml)		*Saprolegnia parasitica*	[106]
Cupressus sempervirens L.	Cypress (Italian)	Several plant parts	MIC 0.75<br>(μg/ml)		*Aeromonas hydrophila*	[135]
		Leaves	(Peg400)<br>MIC 17260.0<br>MBC 17260.0	(DMSO)<br>MIC 34520.0<br>MBC 34520.0	*Aeromonas hydrophila*	[111]
			(% v/v)			
		n/a	MIC >6.4<br>MBC >6.4<br>(mg/ml)		*Aeromonas hydrophila*	[107]
		Leaves	(Peg400)<br>MIC 17260.0<br>MBC 17260.0	(DMSO)<br>MIC 4315.0<br>MBC 4315.0	*Aeromonas jandaei*	[111]
			(% v/v)			
		n/a	MIC >6.4<br>MBC >6.4<br>(mg/ml)		*Citrobacter freundii*	[107]
Cymbopogon citratus (DC.) Stapf	Lemongrass	Leaves	(Peg400)<br>MIC 2185.0<br>MBC 4370.0	(DMSO)<br>MIC 2185.0<br>MBC 4370.0	*Aeromonas hydrophila*	[111]
			(% v/v)			
			(Peg400)<br>MIC 4370.0<br>MBC 4370.0	(DMSO)<br>MIC 4370.0<br>MBC 4370.0	*Aeromonas jandaei*	
			(% v/v)			
Cymbopogon flexuosus (Nees ex Steud.) Will.Watson	Lemongrass (Cochin grass)	Leaves	MIC 0.25<br>MBC 2.0<br>(% v/v)		*Photobacterium damselae (FP4101)*	[136]
			MIC 0.5<br>MBC 4.0<br>(% v/v)		*Edwardsiella tarda (FP5060)*	
			MIC 0.032<br>MBC 0.125<br>(% v/v)		*Edwardsiella tarda (ED47)*	

			MIC 0.032 MBC 0.125 (% v/v)	*Edwardsiella tarda (Yoshida)*	
			MIC 0.063 MBC 0.25 (% v/v)	*Edwardsiella tarda (ED45)*	
			MIC 0.25 MBC 0.5 (% v/v)	*Lactococcus garviae (FP5245)*	
			MIC 0.125 MBC 0.25 (% v/v)	*Streptococcus iniae (FP3287)*	
			MIC 0.063 MBC 0.25 (% v/v)	*Streptococcus iniae (S186)*	
			MIC 0.125 MBC 0.5 (% v/v)	*Streptococcus iniae (S530)*	
			MIC 0.032 MBC 0.125 (% v/v)	*Streptococcus iniae (S131)*	
			MIC 0.125 MBC 0.5 (% v/v)	*Streptococcus parauberis (S124)*	
			MIC 0.032 MBC 0.032 (% v/v)	*Streptococcus parauberis (S527)*	
			MIC 0.016 MBC 0.063 (% v/v)	*Streptococcus parauberis (1466)*	
Cymbopogon nardus(L.) Rendle	Citronella (Grass)	Leaves	MIC_{50} 25.0 MIC_{90} 50.0 (μl/ml)	*Pseudomonas agglomerans*	[105]
			MIC_{50} 50.0 MIC_{90} 100.0 (μl/ml)	*Pseudomonas antarctica*	
			MIC_{50} 50.0 MIC_{90} 100.0 (μl/ml)	*Pseudomonas brassicacearum*	
			MIC_{50} 12.5 MIC_{90} 25 (μl/ml)	*Pseudomonas frederiksbergensis*	
			MIC_{50} 12.5 MIC_{90} 25.0 (μl/ml)	*Pseudomonas koreensis*	
			MIC_{50} 25.0 MIC_{90} 50.0 (μl/ml)	*Pseudomonas lundensis*	

			MIC_{50} 25.0<br>MIC_{90} 50.0<br>(µl/ml)	*Pseudomonas mandelii*	
			MIC_{50} 12.5<br>MIC_{90} 25.0<br>(µl/ml)	*Pseudomonas proteolytica*	
			MIC_{50} 25.0<br>MIC_{90} 50.<br>(µl/ml)	*Pseudomonas synxantha*	
			MIC_{50} 12.5<br>MIC_{90} 25.0<br>(µl/ml)	*Pseudomonas veronii*	
Cymbopogon winterianus Jowitt ex Bor	Citronella (Java)	Aerial parts	MIC 0.488 (µg/ml)	*Edwardsiella* spp. *(Lates calcarifer)*	[137]
			MIC 0.488 (µg/ml)	*Edwardsiella* spp. *(Macrobranchium rosenbergii)*	
			MIC 0.977 (µg/ml)	*Edwardsiella* spp. *(Rana catesbeiana)*	
			MIC 0.488-0.977 (µg/ml)	*Edwardsiella tarda (Clarias gariepinus)*	
			MIC 0.244 (µg/ml)	*Edwardsiella tarda (Tilapia* sp.)	
			MIC 0.244-0.488 (µg/ml)	*Edwardsiella tarda (Monopterus albus)*	
			MIC 0.244-0.488 (µg/ml)	*Edwardsiella tarda (Trichogaster pectoralis)*	
			MIC 0.244 (µg/ml)	*Vibrio* spp. *(Macrobrachium rosenbergii)*	
			MIC 0.244 (µg/ml)	*Vibrio* spp. *(Penaeus monodon)*	
			MIC 0.244 (µg/ml)	*Vibrio* spp. *(Penaeus vannamei)*	
			MIC 0.244 (µg/ml)	*Vibrio* spp. *(Rana catesbeiana)*	
			MIC 0.244 (µg/ml)	*Vibrio* spp. *(Scylla* sp.)	
			MIC 0.488 (µg/ml)	*Photobacterium damselae subsp. damselae (Lates calcarifer)*	
			MIC 0.488 (µg/ml)	*Aeromonas* spp. *(Macrobrachium rosenbergii)*	
			MIC 0.977 (µg/ml)	*Aeromonas* spp. *(Rana catesbeiana)*	
			MIC 0.488 (µg/ml)	*Escherichia coli (Lates calcarifer)*	

			MIC 0.488 (µg/ml)	*Salmonella* spp. *(Macrobrachium rosenbergii)*	
			MIC 0.244 (µg/ml)	*Salmonella* spp. *(Lates calcarifer)*	
			MIC 0.977 (µg/ml)	*Flavobacterium* spp. *(Rana catesbeiana)*	
			MIC 0.244 (µg/ml)	*Pseudomonas* spp. *(Lates calcarifer)*	
			MIC 0.488 (µg/ml)	*Streptococcus* spp. *(Lates calcarifer)*	
Daucus carota L.	Carrot seed	seed	MIC 31.25<br>MBC 31.25<br>(µl/ml)	*Aeromonas hydrophila*	[117]
			MIC 31.25<br>MBC 31.25<br>(µl/ml)	*Edwardsiella tarda*	
			MIC 31.25<br>MBC 62.5<br>(µl/ml)	*Pseudomonas fluorescence*	
			MIC 0.97<br>MBC 3.9<br>(µl/ml)	*Vibrio alginolyticus*	
Echinophora platylobaDC.	Khousharizeh	Aerial parts	MIC >1000<br>MLC >1000<br>(µg/ml)	*Lactococcus garviae*	[108]
Elettaria cardamomum(L.) Maton	Cardamom (Green)	n/a	MIC 2.0 (µl/ml)	*Candida albicans*	[138]
Elwendia persica(Boiss.) Pimenov & Kljukov (referred to as *Bunium persicum*)	Cumin (Black)	Fruits	MIC 8<br>MLC 16<br>(µl/ml)	*Lactococcus garviae*	[108]
Eryngium campestre L.	Eryngo	aerial parts	MIC 1.0<br>MFC 2.0<br>(µg/ml)	*Saprolegnia parasitica*	[106]
Eucalyptus camaldulensisDehnh.	Eucalyptus (camaldulensis)	Leaves	MIC 62.5<br>MBC 125<br>(µl/ml)	*Vibrio parahaemolyticus*	[132]
			MIC 125<br>MBC 500<br>(µl/ml)	*Aeromonas hydrophila*	
			MIC 31.25<br>MBC 32.5<br>(µl/ml)	*Vibrio vulnificus*	
Eucalyptus globulus Labill.	Eucalyptus (globulus) .	Leaves	(Peg400) MIC 18240.0 MBC 18240.0<br>(DMSO) MIC 18240.0 MBC 18240.0<br>(% v/v)	*Aeromonas hydrophila*	[111]

		Aerial parts	MIC 250.0<br>MBC 250.0<br>(μg/ml)	*Lactococcus garviae*	[115]
		Leaves	(Peg400) MIC 18240.0 MBC 18240.0 / (DMSO) MIC 18240.0 MBC 18240.0<br>(% v/v)	*Aeromonas jandaei*	[111]
		Leaves & flowers	MIC 2.5<br>(μl/ml)	*Saprolegnia parasitica*	[139]
		Leaves & flowers	MIC <20.0<br>(μl/ml)	*Aspergillus fumigatus*	
Foeniculum vulgare Mill.	Purple Fennel (Bitter)	n/a	MIC 50<br>(μg/ml)	*Yersinia ruckeri*	[140]
		Dried fruits	MIC_{50} 12.5<br>MIC_{90} 25.0<br>(μl/ml)	*Pseudomonas agglomerans*	[105]
			MIC_{50} 12.5<br>MIC_{90} 50.0<br>(μl/ml)	*Pseudomonas antarctica*	
			MIC_{50} 25.0<br>MIC_{90} 50.0<br>(μl/ml)	*Pseudomonas brassicacearum*	
			MIC_{50} 12.5<br>MIC_{90} 25.0<br>(μl/ml)	*Pseudomonas frederiksbergensis*	
			MIC_{50} 25.0<br>MIC_{90} 50.0<br>(μl/ml)	*Pseudomonas koreensis*	
			MIC_{50} 12.5<br>MIC_{90} 25.0<br>(μl/ml)	*Pseudomonas lundensis*	
			MIC_{50} 25.0<br>MIC_{90} 50.0<br>(μl/ml)	*Pseudomonas mandelii*	
			MIC_{50} 25.0<br>MIC_{90} 50.0<br>(μl/ml)	*Pseudomonas proteolytica*	
			MIC_{50} 12.5<br>MIC_{90} 25.0<br>(μl/ml)	*Pseudomonas synxantha*	
			MIC_{50} 12.5<br>MIC_{90} 25.0<br>(μl/ml)	*Pseudomonas veronii*	
Gaultheria procumbensL.	Wintergreen Partridge Berry	Green leaves	MIC 3.12<br>(μl/ml)	*Aeromonas* spp.	[114]
			MIC 3.12<br>(μl/ml)	*Aeromonas salmonicida*	
			MIC 3.12<br>(μl/ml)	*Enterococcus faecium*	

			MIC 25.0 (µl/ml)	*Pseudomonas fluorescens*	
			MIC 6.25 (µl/ml)	*Yersinia* spp.	
			MIC 6.25 (µl/ml)	*Yersinia ruckeri*	
Glycyrrhiza glabra L.	Liquorice	Leaves	MIC 920 (µg/ml)	*Lactococcus garviae*	[141]
		Roots	MIC >1000 MLC >1000 (µg/ml)	*Lactococcus garviae*	[108]
Illicium verum Hook.f.	Star Anise	n/a	MIC 1.6 MBC 3.2 (mg/ml)	*Aeromonas hydrophila*	[107]
			MIC >6.4 MBC >6.4 (mg/ml)	*Citrobacter freundii*	
Jasminum grandiflorum L.	Jasmine	Flowers	MIC_{50} 11.36 MIC_{90} 15.26 (µl/ml)	*Pseudomonas fluorescens*	[142]
			MIC_{50} 3.18 MIC_{90} 5.24 (µl/ml)	*Candida albicans*	
Juglans regia L.	Common walnut	seeds	MIC 510 (µg/ml)	*Lactococcus garviae*	[141]
Juniperus communis L.	Juniper (Common)	Aerial parts, leaves & cones	MIC 0.57-0.75 (mg/ml)	*Candida albicans*	[143]
Juniperus deltoides R.P.Adams (referred to as *Juniperus oxycedrus L. subsp. deltoides*)	Juniper (Eastern prickly)		MIC 1.15 (mg/ml)	*Candida albicans*	
Juniperus drupacea Labill.	Juniper (Syrian)		MIC 0.75-0.79 (mg/ml)	*Candida albicans*	
Juniperus excelsa M.Bieb.	Juniper (Greek)		MIC 0.85-0.88 (mg/ml)	*Candida albicans*	
Juniperus foetidissima Willd.	Juniper (Stinking)		MIC 0.62-0.97 (mg/ml)	*Candida albicans*	
Juniperus macrocarpa Sm.	Juniper (Largefruited)		MIC 0.83-0.88 (mg/ml)	*Candida albicans*	
Juniperus turbinata Guss.	Juniper (Mediterranean)		MIC 0.5-0.67 (mg/ml)	*Candida albicans*	
Juniperus Sabina L.	Juniper (Sarin)		MIC 0.9-0.98 (mg/ml)	*Candida albicans*	
Kelussia odoratissima Mozaff.	Karafse kouhi	Leaves	MIC >1000 MLC >1000 (µg/ml)	*Lactococcus garviae*	[108]
Kunzea flavescens C.T.White & W.D.Francis	Kunzea (Yellow)	Leaves	MIC 1875.0-2320.0 (µg/ml)	*Aeromonas hydrophila*	[144]

Larix decidua *Mill.*	Larch	Young shoots	MIC 50.0 MBEC 25.0 (µl/ml)	*Pseudomonas aeruginosa (ATCC27853)*	[145]
			MIC 50.0 MBEC 25.0 (µl/ml)	*Pseudomonas aeruginosa*	
			MIC 25.0 MBEC 12.5 (µl/ml)	*Candida albicans*	
Laurus nobilis L.	Bay Laurel	Leaves	MIC 5.0 MBC 5.0 (µl/ml)	*Pseudomonas aeruginosa*	[125]
			MIC 5.0 MFC 5.0 (µg/ml)	*Candida albicans*	
Lavandula angustifolia Mill.	Lavender (angustifolia)	Flowers	MIC_{50} 25.0 MIC_{90} 50.0 (µl/ml)	*Pseudomonas agglomerans*	[105]
			MIC_{50} 50.0 MIC_{90} 100.0 (µl/ml)	*Pseudomonas antarctica*	
			MIC_{50} 50.0 MIC_{90} 100.0 (µl/ml)	*Pseudomonas brassicacearum*	
			MIC_{50} 12.5 MIC_{90} 25.0 (µl/ml)	*Pseudomonas frederiksbergensis*	
			MIC_{50} 12.5 MIC_{90} 25.0 (µl/ml)	*Pseudomonas koreensis*	
			MIC_{50} 12.5 MIC_{90} 25.0 (µl/ml)	*Pseudomonas lundensis*	
			MIC_{50} 25.0 MIC_{90} 50.0 (µl/ml)	*Pseudomonas mandelii*	
			MIC_{50} 25.0 MIC_{90} 50.0 (µl/ml)	*Pseudomonas proteolytica*	
			MIC_{50} 12.5 MIC_{90} 25.0 (µl/ml)	*Pseudomonas synxantha*	
			MIC_{50} 12.5 MIC_{90} 25.0 (µl/ml)	*Pseudomonas veronii*	
Lavandula angustifolia Mill. (not *Lavandula angustifolia Moench* based on Distribution Data of the species and Authors reports that was collected	Lavender (angustifolia)	n/a	MIC 0.125 MBC 0.25 (% v/v)	*Photobacterium damselae (FP4101)*	[146]
			MIC 1.0 MBC 4.0 (% v/v)	*Edwardsiella tarda (FP5060)*	

from Romania (referred to as *Lavendular angustifolia*)			MIC 2.0<br>MBC 8.0<br>(% v/v)	*Edwardsiella tarda (ED47)*	
			MIC 0.5<br>MBC 4.0<br>(% v/v)	*Edwardsiella tarda (Yoshida)*	
			MIC 2.0<br>MBC 8.0<br>(% v/v)	*Edwardsiella tarda (ED45)*	
			MIC 1.0<br>MBC 4.0<br>(% v/v)	*Lactococcus garviae (FP5245)*	
			MIC 0.5<br>MBC 2.0<br>(% v/v)	*Streptococcus iniae (FP5228)*	
			MIC 0.125<br>MBC 4.0<br>(% v/v)	*Streptococcus iniae (S186)*	
			MIC 0.063<br>MBC 0.5<br>(% v/v)	*Streptococcus iniae (S530)*	
			MIC 0.125<br>MBC 1.0<br>(% v/v)	*Streptococcus iniae (S131)*	
			MIC 0.5<br>MBC 2.0<br>(% v/v)	*Streptococcus parauberis (S124)*	
			MIC 0.125<br>MBC 0.5<br>(% v/v)	*Streptococcus parauberis (S527)*	
			MIC 0.25<br>MBC 1.0<br>(% v/v)	*Streptococcus parauberis (1466)*	
Lippia origanoides**Kunth**	Oregano (Mexican)	Leaves	MIC 0.2<br>MBC 0.2<br>(mg/ml)	*Aeromonas hydrophila*	[107]
			MIC 0.8<br>MBC 0.8<br>(mg/ml)	*Citrobacter freundii*	
Lippia origanoidesKunth (referred to as *Lippia sidoides*)	Pepper rosmarin	Inflorescences and leaves	MIC 625.0<br>MFC 625.0<br>(µg/ml)	*Aeromonas hydrophila*	[147]
			MIC 625.0<br>MFC 625.0<br>(µg/ml)	*Aeromonas spp (248)*	
		Leaves	(Peg400) MIC 2245.0 MBC 4490.0; (DMSO) MIC 8980.0 MBC 17960.0<br>(% v/v)	*Aeromonas hydrophila*	[111]

			(Peg400) MIC 17960.0 MBC 17960.0 / (DMSO) MIC 17960.0 MBC 17960.0 (% v/v)	*Aeromonas jandaei*	
Litsea cubeba(Lour.)Pers.	Mountain Pepper	Whole Plant	MIC 12.5 (µl/ml)	*Aeromonas* spp.	[114]
			MIC 12.5 (µl/ml)	*Aeromonas salmonicida*	
			MIC 3.12 (µl/ml)	*Enterococcus faecium*	
			MIC 25.0 (µl/ml)	*Pseudomonas fluorescens*	
			MIC 6.25 (µl/ml)	*Yersinia* spp.	
			MIC 6.25 (µl/ml)	*Yersinia ruckeri*	
Matricaria chamomilla L. (referred to as *Matricaria recutita)*	Chamomile (German)	Leaves & flowers	MIC 5.0 (µl/ml)	*Saprolegnia parasitica*	[139]
			MIC 5.0 (µl/ml)	*Aspergillus fumigatus*	
		n/a	MIC_{50} 11.56 MIC_{90} 13.35 (µl/ml)	*Pseudomonas aeruginosa*	[131]
			MIC_{50} 11.56 MIC_{90} 13.35 (µl/ml)	*Candida albicans*	
Melaleuca alternifolia(Maiden & Betche) Cheel	Tea Tree	Leaves	MIC 3.2 MBC 6.4 (mg/ml)	*Aeromonas hydrophila*	[107]
			MIC >6.4 MBC >6.4 (mg/ml)	*Citrobacter freundii*	
Melaleuca cajuputiMaton & Sm. Ex R.Powell	Cajeput	Leaves	MIC 6.25 MBC 25.0 (mg/ml)	*Vibrio harveyi*	[148
			MIC 6.25 MBC 25.0 (mg/ml)	*Vibrio parahaemolyticus*	
Melaleuca ericifolia Sm.	swamp paperbark	Wood bark	MIC 6.25 (µl/ml)	*Aeromonas* spp.	[114]
			MIC 12.5 (µl/ml)	*Aeromonas salmonicida*	
			MIC 6.25 (µl/ml)	*Enterococcus faecium*	
			MIC 12.5 (µl/ml)	*Pseudomonas fluorescens*	
			MIC 6.25 (µl/ml)	*Yersinia* spp.	
			MIC 12.5 (µl/ml)	*Yersinia ruckeri*	

***Melaleuca leucadendra*(L.)L (referred to as *Melaleuca leucadendron and Melaleuca leucadendra*)**	Paperbark (White Weeping)	Wood bark	MIC 6.25 (µl/ml)	*Aeromonas* spp.	
			MIC 12.5 (µl/ml)	*Aeromonas salmonicida*	
			MIC 6.25 (µl/ml)	*Enterococcus faecium*	
			MIC 12.5 (µl/ml)	*Pseudomonas fluorescens*	
			MIC 12.5 (µl/ml)	*Yersinia* spp.	
			MIC 12.5 (µl/ml)	*Yersinia ruckeri*	
***Melissa officinalis*L.**	Melissa (Leomn Balm)	Aerial parts	MIC 1.0 MBC 2.0 (µl/ml)	*Acinetobacter baumannii (ATCC 19606)*	[149]
			MIC 0.5 MBC 1.0 (µl/ml)	*Pseudomonas aeruginosa (ATCC 25922)*	
			MIC 1.0 MBC 2.0 (µl/ml)	*Staphylococcus aureus (ATCC 9144)*	
***Mentha longifolia*(L.)L.**	Mint (Horsemint)	Leaves & flowers	MIC 10.0 (µl/ml)	*Saprolegnia parasitica*	[139]
			MIC 20.0 (µl/ml)	*Aspergillus fumigatus*	
***Mentha × piperita*L.**	Mint (Peppermint)	Aerial parts	MIC 1.0 MFC 2.0 (µg/ml)	*Saprolegnia parasitica*	[106]
		n/a	MIC_{50} 11.56 MIC_{90} 13.35 (µl/ml)	*Pseudomonas aeruginosa*	[131]
			MIC_{50} 5.56 MIC_{90} 7.23 (µl/ml)	*Candida albicans*	
		Leaves	MIC_{50} 12.5 MIC_{90} 25.0 (µl/ml)	*Pseudomonas agglomerans*	[105]
			MIC_{50} 12.5 MIC_{90} 25.0 (µl/ml)	*Pseudomonas antarctica*	
			MIC_{50} 25.0 MIC_{90} 50.0 (µl/ml)	*Pseudomonas brassicacearum*	
			MIC_{50} 25.0 MIC_{90} 50.0 (µl/ml)	*Pseudomonas frederiksbergensis*	
			MIC_{50} 12.5 MIC_{90} 25.0 (µl/ml)	*Pseudomonas koreensis*	

			MIC_{50} 12.5 MIC_{90} 25.0 (µl/ml)	*Pseudomonas lundensis*	
			MIC_{50} 25.0 MIC_{90} 50.0 (µl/ml)	*Pseudomonas mandelii*	
			MIC_{50} 25.0 MIC_{90} 50.0 (µl/ml)	*Pseudomonas proteolytica*	
			MIC_{50} 12.5 MIC_{90} 25.0 (µl/ml)	*Pseudomonas synxantha*	
			MIC_{50} 12.5 MIC_{90} 25.0 (µl/ml)	*Pseudomonas veronii*	
Mentha spicata L.	Spearmint	Leaves	MIC_{50} 25.0 MIC_{90} 50.0 (µl/ml)	*Pseudomonas agglomerans*	
			MIC_{50} 50.0 MIC_{90} 100.0 (µl/ml)	*Pseudomonas antarctica*	
			MIC_{50} 50.0 MIC_{90} 100.0 (µl/ml)	*Pseudomonas brassicacearum*	
			MIC_{50} 12.5 MIC_{90} 25 (µl/ml)	*Pseudomonas frederiksbergensis*	
			MIC_{50} 12.5 MIC_{90} 25.0 (µl/ml)	*Pseudomonas koreensis*	
			MIC_{50} 12.5 MIC_{90} 50.0 (µl/ml)	*Pseudomonas lundensis*	
			MIC_{50} 6.25 MIC_{90} 12.5 (µl/ml)	*Pseudomonas mandelii*	
			MIC_{50} 12.5 MIC_{90} 25.0 (µl/ml)	*Pseudomonas proteolytica*	
			MIC_{50} 12.5 MIC_{90} 25.0 (µl/ml)	*Pseudomonas synxantha*	
			MIC_{50} 12.5 MIC_{90} 25.0 (µl/ml)	*Pseudomonas veronii*	
Momordica charantia L.	Melon (Bitter Gourd)	Fresh leaves	MIC 1.2 (mg/ml)	*Aeromonas hydrophila*	[109]
			MIC 1.2 (mg/ml)	*Pseudomonas fluorescens*	

<table>
<tr><td></td><td></td><td></td><td colspan="2">MIC 2.5
(mg/ml)</td><td>Edwardsiella tarda</td><td></td></tr>
<tr><td rowspan="3">Monodora myristica(Gaertn.) Dunal</td><td rowspan="3">Nutmeg (African)</td><td rowspan="3">Seeds</td><td colspan="2">MIC 150.0
MBC 150.0
(mg/ml)</td><td>Pseudomonas aeruginosa (AAU2)</td><td rowspan="3">[150]</td></tr>
<tr><td colspan="2">MIC 600.0
MBC 600.0
(mg/ml)</td><td>Pseudomonas aeruginosa (PB112 165)</td></tr>
<tr><td colspan="2">MIC 300.0
MFC 300.0
(mg/ml)</td><td>Aspergillus flavus</td></tr>
<tr><td rowspan="2">Myristica fragransHoutt.</td><td rowspan="2">Nutmeg</td><td rowspan="2">Seeds</td><td colspan="2">MIC >2000.0
(mg/L)</td><td>Pseudomonas aeruginosa</td><td rowspan="2">[151]</td></tr>
<tr><td colspan="2">MIC 2000.0
MFC >2000.0
(mg/L)</td><td>Candida albicans</td></tr>
<tr><td rowspan="2">Myrtus communis L.</td><td rowspan="4">Myrtle (Common)</td><td rowspan="2">Leaves</td><td colspan="2">MIC >1000
MLC >1000
(µl/ml)</td><td>Lactococcus garviae</td><td>[108]</td></tr>
<tr><td colspan="2">MIC 672.0
(µg/ml)</td><td>Lactococcus garviae</td><td>[141]</td></tr>
<tr><td rowspan="2">Myrtus communis L. (not Myrtus communis Blanco -synonym of Decaspermum blancoi- based on Distribution Data of the species and Authors reports that specimen can fe found in Mediterranean region (referred to as Myrtus communis)</td><td rowspan="2">Leaves & flowers</td><td colspan="2">MIC 10.0
(µl/ml)</td><td>Saprolegnia parasitica</td><td rowspan="2">[139]</td></tr>
<tr><td colspan="2">MIC 20.0
(µl/ml)</td><td>Aspergillus fumigatus</td></tr>
<tr><td>Ocimum americanum L.</td><td>Basil (Hoary)</td><td>Inflorescences and leaves</td><td colspan="2">MIC 6400.0
(µg/ml)</td><td>Aeromonas hydrophila</td><td>[152]</td></tr>
<tr><td rowspan="7">Ocimum basilicumL.</td><td rowspan="7">Basil</td><td rowspan="2">n/a</td><td colspan="2">MIC 9.0
(µl/ml)</td><td>Aeromonas veronii</td><td rowspan="2">[153]</td></tr>
<tr><td colspan="2">MIC 3.0
(µl/ml)</td><td>Aeromonas hydrophila</td></tr>
<tr><td rowspan="2">Leaves</td><td>(Peg400)
MIC 8740.0
MBC 8740.0</td><td>(DMSO)
MIC 17480.0
MBC 17480.0</td><td rowspan="2">Aeromonas hydrophila</td><td rowspan="4">[111]</td></tr>
<tr><td colspan="2">(% v/v)</td></tr>
<tr><td rowspan="2">Leaves</td><td>(Peg400)
MIC 17480.0
MBC 17480.0</td><td>(DMSO)
MIC 8740.0
MBC 8740.0</td><td rowspan="2">Aeromonas jandaei</td></tr>
<tr><td colspan="2">(% v/v)</td></tr>
<tr><td>n/a</td><td colspan="2">MIC 9.0
(µl/ml)</td><td>Pseudomonas fluorescens</td><td>[153]</td></tr>
</table>

		Aerial parts	MIC 0.019 MBC >2.5 (μg/ml)	*Aeromonas hydrophila*	[154]
			MIC 0.039 MBC >5.00 (μg/ml)	*Vibrio vulnificus (S5 -Dicentrarchus labrax, Chebba)*	
			MIC 0.019 MBC >2.5 (μg/ml)	*Vibrio vulnificus (V30 -Sparus aurata, Hergla)*	
			MIC 0.039 MBC 10.0 (μg/ml)	*Vibrio parahaemolyticus (S949- Mytilus edulis, Bizerte)*	
			MIC 0.019 MBC 10.0 (μg/ml)	*Vibrio parahaemolyticus* (*S950 -Mytilus edulis, Bizerte)*	
		Leaves	MIC 500 (μg/ml)	*Vibrio parahaemolyticus*	[155]
		Stem	MIC 250 (μg/ml)	*Vibrio parahaemolyticus*	
		Aerial parts	MIC 0.019 MBC >5.0 (μg/ml)	*Vibrio alginolyticus* (*S6 -Dicentrarchus labrax, Chebba)*	[154]
			MIC 0.019 MBC >5.0 (μg/ml)	*Vibrio alginolyticus* (*S7 -Mytilus edulis, Bizerte)*	
			MIC 0.039 MBC >5.0 (μg/ml)	*Vibrio alginolyticus* (*S8 (Sparus aurata, Hergla)*	
			MIC 0.019 MBC >5.0 (μg/ml)	*Listonella anguillarum (Malaga, Spain)*(referred to as *Vibrio anguillarum)*	
			MIC 0.019 MBC >5.0 (μg/ml)	*Vibrio harveyi (ATCC 18293)* (referred to as *Vibrio harveyii)*	
		Leaves	MIC_{50} 12.5 MIC_{90} 25.0 (μl/ml)	*Pseudomonas agglomerans*	[105]
			MIC_{50} 12.5 MIC_{90} 50.0 (μl/ml)	*Pseudomonas antarctica*	
			MIC_{50} 25.0 MIC_{90} 50.0 (μl/ml)	*Pseudomonas brassicacearum*	
			MIC_{50} 12.5 MIC_{90} 25.0 (μl/ml)	*Pseudomonas frederiksbergensis*	
			MIC_{50} 25.0 MIC_{90} 50.0 (μl/ml)	*Pseudomonas koreensis*	

			MIC_{50} 12.5 MIC_{90} 50.0 (µl/ml)	*Pseudomonas lundensis*	
			MIC_{50} 6.25 MIC_{90} 12.5 (µl/ml)	*Pseudomonas mandelii*	
			MIC_{50} 12.5 MIC_{90} 25.0 (µl/ml)	*Pseudomonas proteolytica*	
			MIC_{50} 12.5 MIC_{90} 25.0 (µl/ml)	*Pseudomonas synxantha*	
			MIC_{50} 12.5 MIC_{90} 25.0 (µl/ml)	*Pseudomonas veronii*	
Ocimum gratissimum L.	Basil (Clove)	Inflorescences and leaves	MIC 5000.0 MBC 5000.0 (µg/ml)	*Aeromonas hydrophila*	[147]
			MIC 2500.0 MBC 5000.0 (µg/ml)	*Aeromonas spp (248)*	
Ocimum tenuiflorum L. (rederred to as Ocimum *sanctum*)	Basil (Holy)	Leaves	MIC 240.0 (µg/ml)	*Lactococcus garviae*	[127]
Origanum vulgare L.	Oregano	n/a	MIC_{50} 3.22 MIC_{90} 5.46 (µl/ml)	*Pseudomonas aeruginosa*	[131]
			MIC_{50} 3.22 MIC_{90} 5.46 (µl/ml)	*Candida albicans*	
		Whole Herb	MIC_{50} 6.25 MIC_{90} 21.5 (µl/ml)	*Pseudomonas agglomerans*	[105]
			MIC_{50} 12.5 MIC_{90} 25.0 (µl/ml)	*Pseudomonas antarctica*	
			MIC_{50} 6.25 MIC_{90} 12.5 (µl/ml)	*Pseudomonas brassicacearum*	
			MIC_{50} 12.5 MIC_{90} 25.0 (µl/ml)	*Pseudomonas frederiksbergensis*	
			MIC_{50} 6.25 MIC_{90} 12.5 (µl/ml)	*Pseudomonas koreensis*	
			MIC_{50} 12.5 MIC_{90} 50.0 (µl/ml)	*Pseudomonas lundensis*	

			MIC_{50} 6.25 MIC_{90} 12.5 (µl/ml)	*Pseudomonas mandelii*	
			MIC_{50} 12.5 MIC_{90} 25.0 (µl/ml)	*Pseudomonas proteolytica*	
			MIC_{50} 12.5 MIC_{90} 25.0 (µl/ml)	*Pseudomonas synxantha*	
			MIC_{50} 12.5 MIC_{90} 25.0 (µl/ml)	*Pseudomonas veronii*	
Pandanus amaryllifolius Roxb. Ex Lindl.	Pandan	Leaves	MIC 62.5 (µg/ml)	*Pseudomonas aeruginosa*	[156]
Peganum harmala L.	Harmala (Syrian rue	Leaves	MIC 105 (µg/ml)	*Lactococcus garviae*	[141]
Pelargonium graveolens L'Hér.	Geranium (Rose)	Leaves	(Peg400) MIC 2222.5 MBC 2222.5; (DMSO) MIC 4444.5 MBC 4444.5 (% v/v)	*Aeromonas hydrophila*	[111]
			(Peg400) MIC 4444.5 MBC 4444.5; (DMSO) MIC 4444.5 MBC 4444.5 (% v/v)	*Aeromonas jandaei*	
Persicaria hydropiper (L.) Delarbre (referred to as *Polygonum hydropiper*)	Water pepper	Fresh leaves	MIC 1.2 (mg/ml)	*Aeromonas hydrophila*	[109]
			MIC 1.2 (mg/ml)	*Pseudomonas fluorescens*	
			MIC 2.5 (mg/ml)	*Edwardsiella tarda*	
Persicaria odorata (Lour.) Soják	Coriander (Vietnamese)	Several plant parts	MIC 3.13 (µg/ml)	*Staphylococcus aureus*	[120]
Petroselinum crispum(Mill.)Fuss	Parsley	Aerial parts	MIC 0.022 MFC >11.25 (µg/ml)	*Aeromonas Hydrophila*	[154]
			MIC 0.011 MFC 5.62 (µg/ml)	*Vibrio vulnificus (S5 -Dicentrarchus labrax, Chebba)*	
			MIC 0.011 MFC >5.62 (µg/ml)	*Vibrio vulnificus (V30 -Sparus aurata, Hergla)*	
			MIC 0.022 MFC >11.25 (µg/ml)	*Vibrio parahaemolyticus (S949 -Mytilus edulis, Bizerte)*	
			MIC 0.044 MFC >11.25 (µg/ml)	*Vibrio parahaemolyticus (S950 -Mytilus edulis, Bizerte)*	

			MIC 0.011 MFC >5.62 (µg/ml)	*Vibrio alginolyticus (S6 -Dicentrarchus labrax, Chebba)*	
			MIC 0.011 MFC >11.25 (µg/ml)	*Vibrio alginolyticus* (*S7 -Mytilus edulis, Bizerte)*	
			MIC 0.022 MFC >2.81 (µg/ml)	*Vibrio alginolyticus (S8 -Sparus aurata, Hergla)*	
			MIC 0.011 MFC >11.25 (µg/ml)	*Listonella anguillarum (Malaga, Spain)*(referred to as *Vibrio anguillarum*)	
			MIC 0.011 MFC >11.25 (µg/ml)	*Vibrio harveyi (ATCC 18293)*(referred to as *Vivrio harveyii*)	
Picea abies(L.) H.Karst.	Spruce (Norway)	Young shoots	MIC 50.0 MBEC 25.0 (µl/ml)	*Pseudomonas aeruginosa (ATCC 27853)*	[145]
			MIC 50.0 MBEC 25.0 (µl/ml)	*Pseudomonas aeruginosa*	
			MIC 6.25 MBEC 3.13 (µl/ml)	*Candida albicans*	
Pimpinella affinisLedeb.	Anise (Aniseed)	Aerial parts	MIC 2.0 MFC 4.0 (µg/ml)	*Saprolegnia parasitica*	[106]
Pimpinella anisumL.	Anise (burnet saxifrage)	Fruits	MIC_{50} 12.5 MIC_{90} 25.0 (µl/ml)	*Pseudomonas agglomerans*	[105]
			MIC_{50} 12.5 MIC_{90} 50.0 (µl/ml)	*Pseudomonas antarctica*	
			MIC_{50} 25.0 MIC_{90} 50.0 (µl/ml)	*Pseudomonas brassicacearum*	
			MIC_{50} 12.5 MIC_{90} 25.0 (µl/ml)	*Pseudomonas frederiksbergensis*	
			MIC_{50} 25.0 MIC_{90} 50.0 (µl/ml)	*Pseudomonas koreensis*	
			MIC_{50} 25.0 MIC_{90} 50.0 (µl/ml)	*Pseudomonas lundensis*	
			MIC_{50} 25.0 MIC_{90} 50.0 (µl/ml)	*Pseudomonas mandelii*	

			MIC_{50} 12.5 MIC_{90} 25.0 (µl/ml)	*Pseudomonas proteolytica*	
			MIC_{50} 25.0 MIC_{90} 50.0 (µl/ml)	*Pseudomonas synxantha*	
			MIC_{50} 25.0 MIC_{90} 50.0 (µl/ml)	*Pseudomonas veronii*	
Pinus mugo Turra	Pine (Swiss Mountain)	Needles	MIC_{50} 6.25 MIC_{90} 21.5 (µl/ml)	*Pseudomonas agglomerans*	[105]
			MIC_{50} 12.5 MIC_{90} 25.0 (µl/ml)	*Pseudomonas antarctica*	
			MIC_{50} 6.25 MIC_{90} 12.5 (µl/ml)	*Pseudomonas brassicacearum*	
			MIC_{50} 12.5 MIC_{90} 25.0 (µl/ml)	*Pseudomonas frederiksbergensis*	
			MIC_{50} 6.25 MIC_{90} 12.5 (µl/ml)	*Pseudomonas koreensis*	
			MIC_{50} 6.25 MIC_{90} 12.5 (µl/ml)	*Pseudomonas lundensis*	
			MIC_{50} 6.25 MIC_{90} 12.5 (µl/ml)	*Pseudomonas mandelii*	
			MIC_{50} 6.25 MIC_{90} 12.5 (µl/ml)	*Pseudomonas proteolytica*	
			MIC_{50} 12.5 MIC_{90} 25.0 (µl/ml)	*Pseudomonas synxantha*	
			MIC_{50} 6.25 MIC_{90} 12.5 (µl/ml)	*Pseudomonas veronii*	
Pinus nigra subsp. pallasiana (Lamb.) Holmboe (referred to as *Pinus nigra Arn. sub sp pallasiana*)	Pine (European black pine)	Several plant parts	MIC 0.5 (µg/ml)	*Aeromonas hydrophila*	[135]
		Young shoots	MIC 25.0 MBEC 12.5 (µl/ml)	*Pseudomonas aeruginosa (ATCC27853)*	[145]
			MIC 25.0 MBEC 12.5 (µl/ml)	*Pseudomonas aeruginosa*	
			MIC 6.25 MBEC 3.13 (µl/ml)	*Candida albicans*	

Pinus sylvestris L.	Pine (Scots)	Needles	MIC_{50} 12.5 MIC_{90} 25.0 (µl/ml)	*Pseudomonas agglomerans*	[105]
			MIC_{50} 12.5 MIC_{90} 50.0 (µl/ml)	*Pseudomonas antarctica*	
			MIC_{50} 25.0 MIC_{90} 50.0 (µl/ml)	*Pseudomonas brassicacearum*	
			MIC_{50} 12.5 MIC_{90} 25.0 (µl/ml)	*Pseudomonas frederiksbergensis*	
			MIC_{50} 25.0 MIC_{90} 50.0 (µl/ml)	*Pseudomonas koreensis*	
			MIC_{50} 6.25 MIC_{90} 21.5 (µl/ml)	*Pseudomonas lundensis*	
			MIC_{50} 6.25 MIC_{90} 12.5 (µl/ml)	*Pseudomonas mandelii*	
			MIC_{50} 6.25 MIC_{90} 12.5 (µl/ml)	*Pseudomonas proteolytica*	
			MIC_{50} 12.5 MIC_{90} 25.0 (µl/ml)	*Pseudomonas synxantha*	
			MIC_{50} 6.25 MIC_{90} 12.5 (µl/ml)	*Pseudomonas veronii*	
Piper betle L.	Betel (Sireh)	Leaves	MIC 25.0 MBC 50.0 (mg/ml)	*Aeromonas hydrophila (ATCC 49140)*	[157]
Piper nigrum L.	Black Pepper	Leaves	MIC 12.5 MBC 100.0 (mg/ml)	*Aeromonas hydrophila (ATCC 49140)*	
Referred to as ***Piper sarmentosum*** (Thus indistinguishable between *Piper sarmentosum* Roxb. or *Piper sarmentosum* Wall. (a synonym of *Piper longum*), as both species can be found in Malaisia where specimen purchased)	Pepper (Lolot)	Leaves	MIC 12.5 MBC 100.0 (mg/ml)	*Aeromonas hydrophila (ATCC 49140)*	
**Pistacia lentiscus L.**	Mastic	Leaves	MIC 5.0 MBC 5.0 (µl/ml)	*Pseudomonas aeruginosa*	[125]
			MIC 5.0 MFC 5.0 (µg/ml)	*Candida albicans*	

Pogostemon cablin (Blanco) Benth.	Patchouli	Leaves	MIC 3.12 (µl/ml)	*Aeromonas* spp.	[114]
			MIC 6.25 (µl/ml)	*Aeromonas salmonicida*	
			MIC 6.25 (µl/ml)	*Enterococcus faecium*	
			MIC 12.5 (µl/ml)	*Pseudomonas fluorescens*	
			MIC 12.5 (µl/ml)	*Yersinia* spp.	
			MIC 12.5 (µl/ml)	*Yersinia ruckeri*	
Pseudevernia furfuracea (L.)Zopf.	Tree moss	Whole Lichen	MIC 125.0, 250.0 (µg/ml)	*Aeromonas hydrophila (DSM 30187)*	[158]
			MIC 250.0 (µg/ml)	*Pseudomonas anguiliseptica (DSM 12111)*	
			MIC 125.0 (µg/ml)	*Edwardsiella tarda (DSM 30052)*	
			MIC 250.0, 500.0 (µg/ml)	*Listonella anguillarum (ATCC 19264)*	
			MIC 250.0 (µg/ml)	*Yersinia ruckeri (ATCC 29473)*	
Pseudotsuga menziesii (Mirb.) Franco	Fir (Douglas)	Young shoots	MIC 25.0 MBEC 12.5 (µl/ml)	*Pseudomonas aeruginosa (ATCC27853)*	[145]
			MIC 25.0-50.0 MBEC 12.5-25.0 (µl/ml)	*Pseudomonas aeruginosa*	
			MIC 25.0 MBEC 12.5 (µl/ml)	*Candida albicans*	
Psidium guajava L.	Guava	Fresh leaves	MIC 1.2 (mg/ml)	*Aeromonas hydrophila*	[109]
			MIC 1.2 (mg/ml)	*Pseudomonas fluorescens*	
			MIC 5.0 (mg/ml)	*Edwardsiella tarda*	
Punica granatumL.	Pomegranate	Flowers	MIC >1000.0 MLC >1000.0 (µg/ml)	*Lactococcus garviae*	[108]
Quercus brantiiLindl.	Brant's oak Persian oak	Seed (flour)	MIC >1000.0 MLC >1000.0 (µg/ml)	*Lactococcus garviae*	[108]
		Leaves	MIC 978.0 (µg/ml)	*Lactococcus garviae*	[141]
Salvia officinalisL.	Salvia	Leaves	MIC_{50} 25.0 MIC_{90} 50.0 (µl/ml)	*Pseudomonas agglomerans*	[105]

			MIC_{50} 50.0 MIC_{90} 100.0 (µl/ml)	*Pseudomonas antarctica*	
			MIC_{50} 50.0 MIC_{90} 100.0 (µl/ml)	*Pseudomonas brassicacearum*	
			MIC_{50} 12.5 MIC_{90} 25.0 (µl/ml)	*Pseudomonas frederiksbergensis*	
			MIC_{50} 12.5 MIC_{90} 25.0 (µl/ml)	*Pseudomonas koreensis*	
			MIC_{50} 12.5 MIC_{90} 50.0 (µl/ml)	*Pseudomonas lundensis*	
			MIC_{50} 6.25 MIC_{90} 12.5 (µl/ml)	*Pseudomonas mandelii*	
			MIC_{50} 12.5 MIC_{90} 25.0 (µl/ml)	*Pseudomonas proteolytica*	
			MIC_{50} 12.5 MIC_{90} 25.0 (µl/ml)	*Pseudomonas synxantha*	
			MIC_{50} 12.5 MIC_{90} 25.0 (µl/ml)	*Pseudomonas veronii*	
Salvia rosmarinus Spenn. (referred to as *Rosmarinus officinalis*)	Rosemary	Aerial parts	MIC 15.6 MBC 31.2 (µg/ml)	*Lactococcus garviae*	[115]
		n/a	MIC_{50} 23.45 MIC_{90} 26.15 (µl/ml)	*Pseudomonas aeruginosa*	[131]
			MIC_{50} 3.22 MIC_{90} 5.46 (µl/ml)	*Candida albicans*	
		Whole herb	MIC_{50} 25.0 MIC_{90} 50.0 (µl/ml)	*Pseudomonas agglomerans*	[105]
			MIC_{50} 50.0 MIC_{90} 100.0 (µl/ml)	*Pseudomonas antarctica*	
			MIC_{50} 50.0 MIC_{90} 100.0 (µl/ml)	*Pseudomonas brassicacearum*	
			MIC_{50} 12.5 MIC_{90} 25.0 (µl/ml)	*Pseudomonas frederiksbergensis*	

			MIC_{50} 12.5 MIC_{90} 25.0 (µl/ml)	*Pseudomonas koreensis*	
			MIC_{50} 12.5 MIC_{90} 50.0 (µl/ml)	*Pseudomonas lundensis*	
			MIC_{50} 6.25 MIC_{90} 12.5 (µl/ml)	*Pseudomonas mandelii*	
			MIC_{50} 12.5 MIC_{90} 25.0 (µl/ml)	*Pseudomonas proteolytica*	
			MIC_{50} 12.5 MIC_{90} 25.0 (µl/ml)	*Pseudomonas synxantha*	
			MIC_{50} 12.5 MIC_{90} 25.0 (µl/ml)	*Pseudomonas veronii*	
Salvia sclarea L.	Sage (Clary)	n/a	MIC_{50} 11.56 MIC_{90} 13.34 (µl/ml)	*Pseudomonas aeruginosa*	[131]
			MIC_{50} 1.21 MIC_{90} 3.18 (µl/ml)	*Candida albicans*	
Santalum album L.	Sandalwood (Indian)	Wood bark	MIC 1.56 (µl/ml)	*Aeromonas* spp.	[114]
			MIC 3.12 (µl/ml)	*Aeromonas salmonicida*	
			MIC 6.25 (µl/ml)	*Enterococcus faecium*	
			MIC 25.0 (µl/ml)	*Pseudomonas fluorescens*	
			MIC 25.0 (µl/ml)	*Yersinia* spp.	
			MIC 12.5 (µl/ml)	*Yersinia ruckeri*	
Satureja bachtiarica Bunge	Savory (Bakhtiarian)	Leaves	MIC 126.0 (µg/ml)	*Lactococcus garviae*	[141]
		Leaves & flowers	MIC 5.0 (µl/ml)	*Saprolegnia parasitica*	[139]
			MIC 20.0 (µl/ml)	*Aspergillus fumigatus*	
Satureja hortensisL.	Savory (Summer)	Aerial parts	MIC_{50} 12.5 MIC_{90} 50.0 (µl/ml)	*Pseudomonas agglomerans*	[105]
			MIC_{50} 6.25 MIC_{90} 12.5 (µl/ml)	*Pseudomonas antarctica*	

			MIC_{50} 12.5 MIC_{90} 50.0 (µl/ml)	*Pseudomonas brassicacearum*	
			MIC_{50} 12.5 MIC_{90} 25.0 (µl/ml)	*Pseudomonas frederiksbergensis*	
			MIC_{50} 25.0 MIC_{90} 50.0 (µl/ml)	*Pseudomonas koreensis*	
			MIC_{50} 12.5 MIC_{90} 25.0 (µl/ml)	*Pseudomonas lundensis*	
			MIC_{50} 25.0 MIC_{90} 50.0 (µl/ml)	*Pseudomonas mandelii*	
			MIC_{50} 25.0 MIC_{90} 50.0 (µl/ml)	*Pseudomonas proteolytica*	
			MIC_{50} 12.5 MIC_{90} 25.0 (µl/ml)	*Pseudomonas synxantha*	
			MIC_{50} 12.5 MIC_{90} 25.0 (µl/ml)	*Pseudomonas veronii*	
Stachys lavandulifolia Vahl	Pink cotton lamb's ear	Flowers	MIC >1000.0 MLC >1000.0 (µg/ml)	*Lactococcus garviae*	[108]
Styrax pohlii A.D.C. (referred to as *Styrax ferrugineus*)	Styrax (Strigilia)	Leaves	MIC 2500.0 MFC 5000.0 (µg/ml)	*Candida albicans*	[159]
			MIC 2500.0 MBC 5000.0 (µg/ml)	*Staphylococcus aureus*	
Syzygium aromaticum (L.) Merr. & L.M.Perry	Clove	Leaves	(Peg400) MIC 662.5 MBC 1325.0; (DMSO) MIC 662.5 MBC 1325.0 (% v/v)	*Aeromonas hydrophila*	[111]
		Several plant parts	MIC 12.5 (µg/ml)	*Aeromonas hydrophila*	[120]
		Leaves	(Peg400) MIC 2650.0 MBC 2650.0; (DMSO) MIC 2650.0 MBC 2650.0 (% v/v)	*Aeromonas jandaei*	[111]
		Flower buds	MIC 30.0 MBC 30.0 (µg/ml)	*Lactococcus garviae*	[127]
		Several plant parts	MIC 1.56 (µg/ml)	*Vibrio parahaemolyticus*	[120]
			MIC 3.13 (µg/ml)	*Vibrio vulnificus*	

Tanacetum parthenium (L.) Sch.Bip.	Manzanila Feverfew	Leaves	MIC 824.0 (μg/ml)	*Lactococcus garviae*	[141]
Tamarindus indica L.	Tamarind	Fresh leaves	MIC 5.0 (mg/ml)	*Aeromonas hydrophila*	[109]
			MIC 5.0 (mg/ml)	*Pseudomonas fluorescens*	
			MIC 5.0 (mg/ml)	*Edwardsiella tarda*	
Tetrataenium lasiopetalum (Boiss.) Manden (referred to as *Heracleum lasiopetalum*)	Golpar-e-barfi	Leaves	MIC >1000.0 MLC >1000.0 (μg/ml)	*Lactococcus garviae*	[108]
Teucrium polium L.	Felty germander	Aerial parts	MIC >1000.0 MLC >1000.0 (μl/ml)	*Lactococcus garviae*	
Thymbra spicata L.	Savory (Spiked)	Aerial parts (Inflorescences)	MIC 8.0 MLC 16.0 (μl/ml)	*Lactococcus garviae*	
Thymus daenensis čelak.	Thyme (Denaian)	Aerial Parts (Inflorescences)	MIC 8.0 MLC 16.0 (μl/ml)	*Lactococcus garviae*	
		Leaves & flowers	MIC 5.0 (μl/ml)	*Saprolegnia parasitica*	[139]
			MIC 10.0 (μl/ml)	*Aspergillus fumigatus*	
Thymus eigii (Zohary & P.H.Davis) Jalas	Thyme (West Asia)	Several plant parts	MIC 0.05 (μg/ml)	*Aeromonas hydrophila*	[135]
Thymus serpyllum L.	Thyme (Breckland, creeping)	n/a	MIC_{50} 11.56 MIC_{90} 13.34 (μl/ml)	*Pseudomonas aeruginosa*	[131]
			MIC_{50} 3.22 MIC_{90} 5.46 (μl/ml)	*Candida albicans*	
		Leaves	MIC_{50} 25.0 MIC_{90} 50.0 (μl/ml)	*Pseudomonas agglomerans*	[105]
			MIC_{50} 50.0 MIC_{90} 100.0 (μl/ml)	*Pseudomonas antarctica*	
			MIC_{50} 50.0 MIC_{90} 100.0 (μl/ml)	*Pseudomonas brassicacearum*	
			MIC_{50} 12.5 MIC_{90} 25.0 (μl/ml)	*Pseudomonas frederiksbergensis*	
			MIC_{50} 12.5 MIC_{90} 25.0 (μl/ml)	*Pseudomonas koreensis*	

			MIC_{50} 12.5<br>MIC_{90} 50.0<br>(µl/ml)		*Pseudomonas lundensis*	
			MIC_{50} 6.25<br>MIC_{90} 12.5<br>(µl/ml)		*Pseudomonas mandelii*	
			MIC_{50} 12.5<br>MIC_{90} 25.0<br>(µl/ml)		*Pseudomonas proteolytica*	
			MIC_{50} 12.5<br>MIC_{90} 25.0<br>(µl/ml)		*Pseudomonas synxantha*	
			MIC_{50} 12.5<br>MIC_{90} 25.0<br>(µl/ml)		*Pseudomonas veronii*	
***Thymus vulgaris*L.**	Thyme (European)	Leaves	(Peg400)<br>MIC 294.06<br>MBC 588.13<br>(% v/v)	(DMSO)<br>MIC 294.06<br>MBC 588.13	*Aeromonas hydrophila*	[111]
			(Peg400)<br>MIC 2352.5<br>MBC 2352.5<br>(% v/v)	(DMSO)<br>MIC 2352.5<br>MBC 2352.5	*Aeromonas jandaei*	
		Whole herb	MIC_{50} 6.25<br>MIC_{90} 21.5<br>(µl/ml)		*Pseudomonas agglomerans*	[105]
			MIC_{50} 12.5<br>MIC_{90} 25.0<br>(µl/ml)		*Pseudomonas antarctica*	
			MIC_{50} 6.25<br>MIC_{90} 12.5<br>(µl/ml)		*Pseudomonas brassicacearum*	
			MIC_{50} 12.5<br>MIC_{90} 25.0<br>(µl/ml)		*Pseudomonas frederiksbergensis*	
			MIC_{50} 6.25<br>MIC_{90} 12.5<br>(µl/ml)		*Pseudomonas koreensis*	
			MIC_{50} 12.5<br>MIC_{90} 50.0<br>(µl/ml)		*Pseudomonas lundensis*	
			MIC_{50} 6.25<br>MIC_{90} 12.5<br>(µl/ml)		*Pseudomonas mandelii*	
			MIC_{50} 12.5<br>MIC_{90} 25.0<br>(µl/ml)		*Pseudomonas proteolytica*	
			MIC_{50} 12.5<br>MIC_{90} 25.0<br>(µl/ml)		*Pseudomonas synxantha*	

			MIC_{50} 12.5<br>MIC_{90} 25.0<br>(µl/ml)	*Pseudomonas veronii*	
		n/a	MIC 40.0<br>(µg/ml)	*Yersinia ruckeri*	[140]
Trachyspermum ammi (L.) Sprague	Ajowan	seeds	MIC 0.04<br>MFC 0.1<br>(µl/ml)	*Fusarium* sp.	[160]
			MIC 0.043<br>MFC 0.07<br>(µl/ml)	*Saprolegnia parasitica*	
Trachyspermum ammi (L.) Sprague referred to as *Trachyspermum copticum*)	Ajowan (Carum-bishop's weed)	Leaves	MIC 453.0<br>(µg/ml)	*Lactococcus garviae*	[141]
Zataria multifloraBoiss.	Thyme (Shirazi)	Aerial parts (Infloresce nces)	MIC 4.0<br>MLC 8.0<br>(µl/ml)	*Lactococcus garviae*	[108]
		Aerial parts	MIC 7.8<br>MBC 15.6<br>(µg/ml)	*Lactococcus garviae*	[115]
			MIC 0.12<br>MBC 0.12<br>(µl/ml)	*Lactococcus garviae*	[112]
Zingiber officinaleRoscoe	Ginger	Rhizome	MIC 31.25<br>MBC 62.5<br>(µl/ml)	*Aeromonas hydrophila*	[132]
			MIC 5000.0<br>MFC 5000.0<br>(µg/ml)	*Aeromonas hydrophila*	[147]
			MIC 2500.0<br>MFC 5000.0<br>(µg/ml)	*Aeromonas spp (248)*	
			MIC 120.0<br>(µg/ml)	*Lactococcus garviae*	[127]
			MIC 2.0<br>MBC 2.0<br>(% v/v)	*Lactococcus garviae (FP5245)*	[161]
			MIC 4.0<br>MBC 8.0<br>(% v/v)	*Photobacterium damselae (FP4101)*	
			MIC 31.25<br>MBC 125<br>(µl/ml)	*Vibrio parahaemolyticus*	[132]
			MIC 31.23<br>MBC 31.25<br>(µl/ml)	*Vibrio vulnificus*	
		n/a	MIC 2.0<br>(µl/ml)	*Candida albicans*	[138]

Table S3. In vitro efficacy of different essential oils and their major compounds for different fish species. Plant and Animal nomenclature in this work follows the Plants of the World Online (POWO) and World Register of Marine Species (WoRMS) databases (accessed on April 6th, 2025). If a species name cited in a reference, differs from, or is inconsistent with the updated POWO & WoRMS taxonomy, the referenced name is retained and noted accordingly. Efficacy (%): The percentage reduction of a microorganism's population. 100- indicates full inhibition, 0-indicates no inhibition. NOTE: in some instances instead of Efficacy (%), there are EC_{50} or LC_{50} values. Values are shown as reported in the source studies. As a quide, for aqueous exposures, 1 ppm≈1 mg·L^{-1}; for dietary inclusion, 1% w/w = 10 g·kg^{-1} feed; for EO volumes, 1% v/v = 10 mL·kg^{-1} feed. Where conversion would require assumptions (e.g., density, chemotype), no transformation is applied

Parasite Species	*Essential Oil* /Active Substance	Concentrations	Exposure Time	Efficacy (%)	*Aquatic Animal Species*	Citation
Anacanthorus spathulatusThatcher & Kayton, 1979	***Minthostachys mollis***(Benth.) Griseb.	80mg/ml	2h 50min	100	***Piaractus brachypomus***(Cuvier, 1818)	[176]
		200mg/ml	2h	100		
		400mg/ml	1h	100		
		600mg/ml	7min	100		
		1000mg/ml	3min	100		
	Origanum vulgareL.	1500mg/ml	4min	100		
		1000mg/ml	8min	100		
		800mg/ml	25min	100		
		600mg/ml	40min	100		
		400mg/ml	1h	100		
		200mg/ml	1h 50min	100		
		80mg/ml	3h	100		
	Salvia rosmarinusSpenn.	1500mg/ml	8min	100		
		1000mg/ml	20min	100		
		800mg/ml	1h 5min	100		
		600mg/ml	1h 15min	100		
		400mg/ml	1h 50min	100		
		200mg/ml	2h 55min	100		
		80mg/ml	4h	100		
***Argulus* sp.**Müller O.F., 1785	***Azadirachta indica***A.Juss(Azadirachtin)	75mg/L	15h	100	***Carassius auratus*** Linnaeus 1758	[177]
		100mg/L	12h	100		
	Curcuma longa L.	50ppm	18h	100		[178]
		100ppm	6h	100		

		200ppm	3h	100		
	Pellitorine	5mg/ml	12h	80		[179]
Argulus foliaceus(Linnaeus, 1758)	***Madhuca longifolia* var. *latifolia***(Roxb.) A.Chev. (referred to as *Madhuca latifolia*)	25mg/L	36h	100	***Cyprinus carpio***Linnaeus 1758	[180]
Cichlidogyrus tilapiaePaperna, 1960	***Carica papaya*** L.	2ml/L	50min	100	***Oreochromis niloticus***Linnaeus 1758	[181]
Clinostomatopsis intermedialis Lamont, 1920 Lunaschi & Drago, 2009 (referred to as *Clinostomum phalacrocoracis*)	***Verbesina alternifolia***(L.) Britton ex Kearney	400ppm	24h	EC_{50}	***Coptodon zillii*** Gervais, 1848 Referred to as *Tilapia zillii*)	[182]
Cryptocaryon irritansBrown, 1951	Nerolidol	10μl/L	30min	100	***Trachinotus ovatus*** Linnaeus 1758	[183]
***Dactylogyrus* sp.**Diesing, 1850	***Allium cepa*** L.	0.4-1.8mg/ml	15min	100	***Oreochromis niloticus***Linnaeus 1758	[184]
	Allium sativumL.	0.02-0.18mg/ml	5min	100		
	Mentha x piperitaL.	1μl/ml	15min	100	***Cyprinus carpio***Linnaeus 1758	[185]
		2.5μl/ml	8min	100		
		5μl/ml	7min	100		
		10μl/ml	2min	100		
	Citrus × limon (L.) Osbeck	1μl/ml	10min	100		
		2.5μl/ml	5min	100		
		5μl/ml	4min	100		
		10μl/ml	3min	100		
	Melaleuca alternifolia (M aiden & Betche) Cheel	0.5μl/ml	3min	100		
		1μl/ml	2min	100		
		2.5μl/ml	1min	100		
Electrotaenia malapteruri (Fritsch, 1886) Nybelin, 1942 (referred to as*Electrotaenia* sp. but according to WoRMS there is only one direct species within the genus)	***Aframomum melegueta***K.Schum.(referred to as *Afranamum melequeta*)	68.36mg/L	1.5min	98.8	***Clarias gariepinus***Burchell 1822	[186]
Enteromyxum leei(Diamant, Lom & Dyková, 1994)	Citral	100μg/ml	6h	20	***Paralichthys olivaceus***Temminck & Schlegel, 1846	[187]
		100μg/ml	12h	85		
		250μg/ml	6h	60		
		250μg/ml	12h	100		
***Gyrodactylus* sp.**von Nordmann, 1832	***Azadirachta indica***A.Juss(Azadirachtin)	25mg/L	4h	100	***Carassius auratus*** Linnaeus 1758	[177]
		30mg/L	3h	100		

Gyrodactylus kobayashiikobayashii Hukuda, 1940	***Curcuma longa*** L.	12mg/L	24h	100	***Carassius auratus*** Linnaeus 1758	[188]
	Cymbopogon martini (Roxb.)Will Watson (Palmarosa oil) (referred to as *Cymbopogon martinii*)	10mg/L	24h	100		
Ichthyophthirius multifiliisFouquet, 1876	***Azadirachta indica***A.Juss(Azadirachtin)	20-40mg/L	12h	100	***Carassius auratus*** Linnaeus 1758	[177]
		50mg/L	6h	100		
	Carica papaya L.	200mg/L	6h	100	***Carassius auratus*** Linnaeus 1758(referred to as *Carassius auratus auratus*)	[189]
	Macleaya cordata(Willd.) R.Br. (sanguinarine)	0.7mg/L	4h	100	***Ctenopharyngodon idella***Valenciennes 1844	[190]
	Macleaya microcarpa(Maxim.) Fedde (dihydrosanguinarine)	7mg/L	4h	100	***Squaliobarbus curriculus***Richardson 1846	[191]
		10mg/L	4h	100		
	Mucuna pruriens (L.) DC.	150mg/L	6h	100	***Carassius auratus*** Linnaeus 1758(referred to as *Carassius auratus auratus*)	[189]
	Sophora flavescensAiton (sophoraflavanone)	4mg/L	3.3min	100	***Ctenopharyngodon idella***Valenciennes 1844	[192]
		1mg/L	16min	100		
Ichthyophthirius multifiliis (protomonts)	***Magnolia officinalis***Rehder & E.H.Wilson (Mangolol)	0.8mg/L	6h	100	***Carassius auratus*** Linnaeus 1758	[193]
	Zingiber officinaleRoscoe (10-gingerol)	8mg/L	4h	100	***Ctenopharyngodon idella***Valenciennes, 1844(referred to as *Ctenopharyngodon idellus*)	[194]
Ichthyophthirius multifiliis (tomonts)	***Magnolia officinalis*** Rehder & E.H.Wilson(Mangolol)	1.0mg/L	6h	100	***Carassius auratus*** Linnaeus 1758	[193]
		10mg/L	20h	91,3		[195]
		20mg/L	20h	100		
	Rhus chinensisMill.(pentagalloylglucose extracted from galls) (referred to as *Gall chinensis*)	80mg/L (fresh solution)	4h	100	***Ictalurus punctatus***Rafinesque 1818	[196]
	Sophora alopecuroides L.	320mg/L	20h	100	***Carassius auratus*** Linnaeus 1758	[195]
	Zanthoxylum asiaticum(L.) Appelhans, Groppo & J.Wen (Chelerythrine) (referred as *Toddalia asiatica*)	1.2mg/L	4h	100		[197]
		3.5mg/L	4h	100		
	Zingiber officinale Roscoe (10-gingerol)	16mg/L	4h	100	***Ctenopharyngodon idella***Valenciennes, 1844(referred to as *Ctenopharyngodon idellus*)	[194]
Ichthyophthirius multifiliis (trophonts)	***Allium cepa*** L.	0.25ml/L	1h	≈94	***Silurus glanis*** Linnaeus 1758	[198]
		0.5ml/L	1h	94		

	Allium sativum L.	0.5ml/L	1h	92		
	Lavandula angustifolia subsp. Angustifolia Mill. (referred as *Lavandula officinalis)*	0.25ml/L	1h	100		
		0.5ml/L	1h	100		
	Mentha spicata L.(referred as *Menthe spicata*)	0.5ml/L	1h	94		
	Origanum onites L.	0,1ml/L	1h	100		
		0.25ml/L	1h	100		
		0.5ml/L	1h	100		
	Salvia officinalis L.	0.5ml/L	1h	100		
Ichthyophthirius multifiliis (theronts)	***Zingiber officinale*** Roscoe (10-gingerol)	2mg/L	4h	100	***Ctenopharyngodon idella*** Valenciennes, 1844(referred to as *Ctenopharyngodon idellus*)	[194]
	Rhus chinensis Mill.(pentagalloylglucose extracted from galls) (referred to as *Gall chinensis*)	20mg/L (fresh solution)	5.6min (± 1.0)	100	***Ictalurus punctatus*** Rafinesque 1818	[196]
		10mg/L (fresh solution)	11.6min (± 1.4)	100		
		5mg/L (fresh solution)	2h 45.min (± 5.2)	100		
		2.5mg/L (fresh solution)	3h 48min (± 4.5)	100		
	Aquilaria sinensis (Lour.) Spreng.	10mg/L	4h	61.3	***Carassius auratus*** Linnaeus 1758	[195]
	Areca catechu L.	10mg/L	4h	10.3		
	Asparagus cochinchinensis (Lour.) Merr.	10mg/L	4h	20.3		
	Bassia scoparia(L.)Voss(referred to as *Kochia scoparia*)	10mg/L	4h	39.3		
	Citrus medica L.	10mg/L	4h	67.7		
	Combretum indicum(L.)DeFilipps(referred as *Quisqualis indica*)	10mg/L	4h	78.3		
	Croton tiglium L.	10mg/L	4h	78.3		
	Dictamnus dasycarpus Turcz.	10mg/L	4h	51		
	Eclipta prostrata (L.)L.	10mg/L	4h	88.3		
	Foeniculum vulgare Mill.(referred to as *Foeniculum uulgare*)	10mg/L	4h	72.3		
	Gardenia jasminoides J.Ellis	10mg/L	4h	71		
	Gentiana manshurica Kitag.	10mg/L	4h	25.3		
	Geranium wilfordii Maxim.	10mg/L	4h	16.7		
	Glycyrrhiza uralensis Fisch. Ex DC.	10mg/L	4h	70.3		

	Hansenia forbesii(H.Boissieu)Pimenov & Kljuykov(referred to as *Notopterygium forbesii*)	10mg/L	4h	79.3		
	Isatis tinctoria subsp. Tinctoria(referred as *Isatis indigotica*)	10mg/L	4h	26.7		
	Kitagawia praeruptora (Dunn)Pimonev (referred to as *Peucedanum praeruptorum*)	10mg/L	4h	35.3		
	Lindera aggregata (Sims) Kosterm.	10mg/L	4h	78		
	Lonicera macrantha(D.Don)Spreng. (referred to as *Lonicera japonica*)	10mg/L	4h	15.3		
	Lycium chinense Mill.	10mg/L	4h	81		
	Magnolia officinalis Rehder & E.H.Wilson	10mg/L	3h	100		
		10mg/L	4h	100		
	Magnolia officinalis Rehder & E.H.Wilson(Mangolol)	0.6mg/L	4h	100	***Carassius auratus*** Linnaeus 1758	[193]
	Mentha canadensisL.(referred to as *Mentha haplocalyx*)	10mg/L	4h	34.7		[195]
	Nelumbo nucifera Gaertn.	10mg/L	4h	60.3		
	Ophiopogon bodinieriH.Lév.	10mg/L	4h	80.7		
	Piper kadsura (Choisy) Ohwi	10mg/L	4h	59.3		
	Rosa laevigata Michx.(referred to as *Rosa laevilgata*)	10mg/L	4h	18.3		
	Scutellaria baicalensisGeorgi	10mg/L	4h	17.3		
	Sinapis albaL.	10mg/L	4h	69.7		
	Sophora alopecuroidesL.	10mg/L	4h	100		
	Trichosanthes KirilowiiMaxim.	10mg/L	4h	88.7		
Mymarothecium viatorumBoeger, Piasecki & Sobecka, 2002	***Minthostachys mollis*** (Benth.) Griseb.	80mg/ml	2h 50min	100	***Piaractus brachypomus***Cuvier 1818	[176]
		200mg/ml	2h	100		
		400mg/ml	1h	100		
		600mg/ml	7min	100		
		1000mg/ml	3min	100		
	Origanum vulgareL.	80mg/ml	3h	100		
		200mg/ml	1h 50min	100		
		400mg/ml	1h	100		
		600mg/ml	40min	100		

		800mg/ml	25min	100		
		1000mg/ml	8min	100		
		1500mg/ml	4min	100		
	Salvia rosmarinus Spenn.	80mg/ml	4h	100		
		200mg/ml	2h 55min	100		
		400mg/ml	1h 50min	100		
		600mg/ml	1h 15min	100		
		800mg/ml	1h 5min	100		
		1000mg/ml	20min	100		
		1500mg/ml	8min	100		
Neobenedenia melleni (MacCallum, 1927) Yamaguti, 1963	***Lippia origanoides*** Kunth	600mg/L	1min	100	***Mugil liza*** *Valenciennes 1836*	[199]
	Lippia origanoides Kunth (referred to as *Lippia sidoides)*	700mg/L	4min	100		
	Mentha x piperita L.	700mg/L	9min	100		
Neoechinorhynchus (Neoechinorhynchus) buttnerae Golvan, 1956	***Aloysia citrodora*** Paláu (referred to *as Aloysia triphylla*)	3.35mg/mL	2h	EC_{50}	***Colossoma macropomum*** Cuvier 1816	[200]
		1.88mg/mL	6h	EC_{50}		
	Croton cajucara Benth.(red morphotype)	3.51mg/mL	2h	EC_{50}		
		1.97mg/mL	6h	EC_{50}		
		1.03mg/mL	24h	EC_{50}		
	Croton cajucara Benth.(white morphotype)	3.51mg/mL	2h	EC_{50}		
		2.72mg/mL	6h	EC_{50}		
		1.49mg/mL	24h	EC_{50}		
	Curcuma longa L.	37.89mg/mL	2h	EC_{50}		
Neoechinorhynchus (Neoechinorhynchus) buttnerae (referred to as *Neoechinorhyncus buttnerae*)	***Dysphania ambrosioides*** (L.) Mosyakin & Clemants(referred to as *Chenopodium ambrosioides*)	10% dried extract/ml	30min	100	***Colossoma macropomum*** Cuvier 1816	[201]
		5% dried extract/ml	15min	96,7		
Neoechinorhynchus (Neoechinorhynchus) buttnerae Golvan, 1956	Eugenol	1.01mg/mL	2h	LC_{50}	***Colossoma macropomum*** Cuvier 1816	[202]
		0.79mg/mL	6h	LC_{50}		
		0.41mg/mL	24h	LC_{50}		
	Lippia grata Schauer (referred to as *Lippia gracilis* Schauer)	1.38mg/mL	2h	EC_{50}		[200]
		0.96mg/mL	6h	EC_{50}		
		0.86mg/mL	24h	EC_{50}		
	Lippia origanoides Kunth	1.20mg/mL	2h	EC_{50}		

		0.86mg/mL	6h	EC_{50}		
		0.76mg/mL	24h	EC_{50}		
	Mentha arvensis L.	0.66mg/mL	24h	EC_{50}		
	Ocimum gratissimum L.	3.51mg/mL	2h	EC_{50}		
		1.97mg/mL	6h	EC_{50}		
		1.03mg/mL	24h	EC_{50}		
	Piper aduncum L.	0.73mg/mL	2h	EC_{50}		
		1.52mg/mL	6h	EC_{50}		
		1.52mg/mL	24h	EC_{50}		
	Tannic acid	4.68mg/mL	2h	LC_{50}		[202]
		1.79mg/mL	6h	LC_{50}		
		1.30mg/mL	24h	LC_{50}		
	Thymol	1.97mg/mL	2h	LC_{50}		
		0.96mg/mL	6h	LC_{50}		
		0.92mg/mL	24h	LC_{50}		
Procamallanus* sp.Baylis, 1923*	***Aframomum meleguetra***K.Schum.(referred to as *Afranamum melequeta*)	68.36mg/L	6.00min	98.2	***Clarias gariepinus***Burchell 1822	[186]
	Azadirachta indicaA.Juss(Azadirachtin)	319.38mg/L	6.5min	95.2		
Salmincola salmoneus (Linnaeus, 1758) (referred to as *Lernaea cyprinacea*)	***Illicium verum*** Hook.f.	12.5μg/ml	2h	EC_{50}	***Carassius auratus*** Linnaeus 1758	[203]
		25μg/ml	1h	EC_{50}		
Schyzocotyle acheilognathi(Yamaguti, 1934) Brabec, Waeschenbach, Scholz, Littlewood & Kuchta, 2015(referred to as *Bothriocephalus acheilognathi*)	***Dianthus chinensis***L.	10g/100ml	90min	100	***Cyprinus carpio***Linnaeus 1758	[204]
		20g/100ml	45min	100		
Sparicotyle chrysophrii(Van Beneden & Hesse, 1863) Mamaev, 1984	Cedrol	0.11mM	4h	EC_{50}	***Sparus aurata***Linnaeus 1758	[205]
	Curcumin	0.24mM	4h	EC_{50}		
	Eucalyptol	0.67mM	4h	EC_{50}		
	Garlicin 80%	0.6mM	4h	EC_{50}		
	1R- Camphor	0.91mM	4h	EC_{50}		
	(+)-trans-Chrysanthemic acid	0.84mM	4h	EC_{50}		
Tenuisentis niloticus (Meyer, 1932) Van Cleave, 1936 (referred to as *Tenuisentis* sp. but according to	***Aframomum meleguetra***K.Schum.(referred to as *Afranamum melequeta*)	68.36mg/L	3.75min	96.5	***Clarias gariepinus***Burchell 1822	[186]

WoRMS there is only one direct species within the genus)	***Azadirachta indica*** A.Juss	319.38mg/L	2.75min	97.4		
***Trichodina* sp.** Ehrenberg, 1830	***Syzygium aromaticum***(L.) Merr. & L.M.Perry	130ppm	60min	100	***Lates calcarifer*** Bloch 1790	[206]
***Wenyonia* sp.** Woodland, 1923	***Aframomum melegueta*** K.Schum.(referred to as *Afranamum melequeta*)	68.36mg/L	5.75min	95.6	***Clarias gariepinus*** Burchell 1822	[186]
	Azadirachta indica A.Juss	319.38mg/L	3.75min	97.1		
Zeylanicobdella arugamensis Silva, 1963	***Curcuma longa*** L.	25mg/ml	10.5min	100	*Epinephelus fuscoguttatus x Epinephelus lanceolatus*	[207]
		50mg/ml	8.06min	100		
		100mg/ml	6.12min	100		
	Senna alata(L.) Roxb.	100mg/ml	≈2.38min	100		[208]

Table S4. *In vivo* efficacy (or EC_{50} values, depending on reported data) of different EOs and their major compounds for different fish species. Plant and Animal nomenclature in this work follows the Plants of the World Online (POWO) and World Register of Marine Species (WoRMS) databases (accessed on April 6th, 2025). If a species name cited in a reference, differs from, or is inconsistent with the updated POWO & WoRMS taxonomy, the referenced name is retained and noted accordingly. **Efficacy (%):** The percentage reduction of a microorganism's population. 100- indicates full inhibition, 0-indicates no inhibition. Efficacy of inhibition is reported with concentrations either per volume or per parts, according to the data reported and based on the application method (e.g., feed or solution dipping).

Parasite Species	*Essential Oil* /Active Substance	Exposure	Concentrations	Efficacy (%)- EC_{50}	*Aquatic Animal Species*	Citation
Anacanthorus spathulatusThatcher & Kayton 1979	***Carapa guianensis*** Aubl.	1h bath/day, for 5 days	500mg/L	91,4	***Colossoma macropomum***Cuvier 1816	[209]
	Copaifera epunctata Amshoff (Oleoresin)(referred to as *Copaifera reticulata*)	1h bath/day, for 3 days	100mg/L	48.5		[210]
	Piper hispidum Sw.	1h bath/d (3 baths -48h Interval between baths)	100mg/L	78.6		[211]
	Piper marginatum Jacq.	20min bath/d (3 baths -48h Interval between baths)	100mg/L	42,8		[212]
***Argulus* sp.**Müller O.F. 1785	***Azadirachta indica***A.Juss(Azadirachtin)	9h	≈62mg/L	EC_{50}	***Carassius auratus*** Linnaeus 1758	[177]
		12h	≈37mg/L	EC_{50}		
		15h	≈28mg/L	EC_{50}		
	Curcuma longaL.	72h	12.5ppm	62.8		[178]
		48h	12.5ppm	≈56		
Argulus coregoniThorell 1865	***Artemisia* sp.**L.	5min	Diping in 1.8 to 3.6 mg/L (not speficied concentration of active compounds)	100	***Cyprinus carpio***Linnaeus 1758	[213]
Argulus foliaceusLinnaeus 1758	***Madhuca longifolia* var. *latifolia***(Roxb.) A.Chev(referred to as *Madhuca latifolia*)	96h	16.45 mg/L	EC_{50}	***Cyprinus carpio***Linnaeus 1758	[180]
Centrocestus formosanus(Nishigori, 1924) Price, 1932	***Azadirachta indica***A.Juss	long bath, 7 days	3g/L	reduced intensity	***Oreochromis niloticus***Linnaeus 1758	[214]
DactylogyridaeBychowsky 1933	***Mentha × villosa*** Huds.	1h	20ml/L	73,5	***Oreochromis urolepis***Norman 1922 (referred to as *Oreochromis hornorum*)	[215]
		1h	20ml/L	79	***Oreochromis aureus***Steindachner, 1864	
		1h	20ml/L	80,7	***Oreochromis mossambicus***Peters 1852	
		1h	20ml/L	84,5	***Oreochromis***	

***Dactylogyrus* sp.**Diesing 1850	***Allium sativum*** L.	72h	0.08g/L ($^1/_5$ of 96h- LC_{50})	85.7	***niloticus***Linnaeus 1758	[210][184]
		72h	0.04g/L ($^1/_{10}$ of 96h- LC_{50})	64.7		
	Allium cepa L.	72h	0.708g/L ($^1/_5$ of 96h- LC_{50})	100.0		
		72h	0.354g/L ($^1/_{10}$ of 96h- LC_{50})	90.5		
	Azadirachta indicaA.Juss	long bath, 7 days	3g/L	reduced intensity		[214]
	Azadirachta indicaA.Juss(Azadirachtin)	2h	≈23mg/L	EC_{50}	***Carassius auratus*** Linnaeus 1758	[177]
		3h	≈15mg/L	EC_{50}		
		4h	≈5mg/L	EC_{50}		
Dactylogyrus intermediusWegener 1910	Cinnamaldehyde	48h	5mg/L	100		[216]
	Cinnamic acid	48h	16mg/L	100		
Gyrodactylus kobayashiiHukuda 1940	***Neolitsea cassia***(L.) Kosterm. (referred to as *Cinnamomum cassia*)	24h	14mg/L	94.57		[188]
	Citrus x limon (L.) Osbeck	24h	10mg/L	5.62		
	Curcuma longaL. (Curcuma oil)	2h	3.48mg/	EC_{50}		
		24h	5.72mg/	EC_{50}		
		24h	12mg/L	100		
	Curcuma zedoaria(Christm.) Roscoe	24h	15mg/L	100		
	Cymbopogon martini (Roxb.)Will Watson (Palmarosa oil) (referred to as *Cymbopogon martinii*)	2h	9.87mg/L	EC_{50}		
		24h	4.98mg/L	EC_{50}		
		24h	10mg/L	100		
	Eucalyptus globulus Labill.	24h	100mg/L	90.84		
	Foeniculum vulgare Mill.	24h	40mg/L	10.10		
	Melaleuca alternifolia (Maiden & Betche) Cheel	24h	40mg/L	100		
	Melia azedarachL. (referred to as *Melia azedarace*)	24h	40mg/L	100		
	Mentha canadensis L. (referred to as *Mentha sachalinensis*)	24h	40mg/L	10.45		
	Nigella sativa L.(Thymoquinone (1,4-benzoquinone)	48h	0.303 mg/L	EC_{50}		[217]
	Origanum vulgare L.	24h	10mg/L	23.36		[188]
	Pimpinella anisumL.	24h	10mg/L	21.92		
	Pogostemon cablin (Blanco) Benth.	24h	10mg/L	100		
	Ruta graveolens L.	24h	25mg/L	100		

	Syzygium aromaticum(L.) Merr. & L.M.Perry	24h	16mg/L	45.06		
	Syzygium aromaticum(L.) Merr. & L.M.Perry(leaf extract)	24h	18mg/L	36.02		
Haplorchis taichui(Nishigori, 1924) Witenberg 1930	***Eurycoma longifolia*** Jack	12h	400mg/ml	100	cyprinid fish	[218]
	Thunbergia laurifoliaLindl.	12h	400mg/ml	100		
	Allium sativum L.	12h	5mg/ml	100		
		3h	20mg/ml	100		
		4 days bath	0.1g/L	100	***Poecilia reticulata***Peters 1859	[219]
Ichthyophthirius multifiliisFouquet 1876	***Azadirachta indica***A.Juss(Azadirachtin)	3h	≈47mg/L	EC_{50}	***Carassius auratus*** Linnaeus 1758	[177]
		4h	≈38mg/L	EC_{50}		
		6h	≈32mg/L	EC_{50}		
		9h	≈23mg/L	EC_{50}		
		12h	≈13mg/L	EC_{50}		
	Carica papaya L.	72h	200mg/L	90	***Carassius auratus*** Linnaeus 1758 *(referred as Carassius auratus auratus)*	[189]
	Macleaya cordata(Willd.)R.Br.(sanguinarine)	48h	0.9mg/L	96,8	***Ctenopharyngodon idella***Valenciennes 1844	[190]
	Macleaya microcarpa(Maxim.) Fedde (dihydrosanguinarine)	48h	5.18mg/L	100	***Squaliobarbus curriculus***Richardson 1846	[191]
		48h	9.43mg/L	100		
	Matricaria chamomilla L.	6 days bath	0.4g/L	100	***Poecilia reticulata***Peters 1859	[219]
		1h/day, 4days	50 µl/L	94.8	***Rhamdia quelen*** Quoy & Gaimard, 1824	[220]
	Mucuna pruriens(L.) DC.	96h	250mg/L	90	***Carassius auratus*** Linnaeus 1758 *(referred as Carassius auratus auratus)*	[189]
Lernaea cyprinacea Linnaeus 1758	***Artemisia* sp.**L.	5min	Dipping in 60% Artemisia Extract Solution	100	***Cyprinus carpio***Linnaeus 1758	[213]
Linguadactyloides brinkmanniThatcher & Kritsky 1983	***Piper hispidum*** Sw.	1h bath/d (3 baths -48h Interval between baths)	100mg/L	78.6	***Colossoma macropomum***Cuvier 1816	[212]
	Piper marginatum Jacq.	20min bath/d (3 baths -48h Interval between baths)	100mg/L	42,8		
Mymarothecium boegeriCohen & Kohn 2005	***Carapa guianensis*** Aubl.	1h bath/day, for 5 days	500mg/L	91,4		[209]

	Copaifera epunctata Amshoff (Oleoresin)(referred to as *Copaifera reticulata*)	1h bath/day, for 3 days	100mg/L	48.5		[210]
	Piper hispidum Sw.	1h bath/d (3 baths -48h Interval between baths)	100mg/L	78.6		[212]
	Piper marginatum Jacq.	20min bath/d (3 baths -48h Interval between baths)	100mg/L	42,8		
Notozothecium janauachenseBelmont-Jégu, Domingues & Martins 2004(referred to as *Notozothecium janauachensis*)	***Carapa guianensis*** Aubl.	1h bath/day, for 5 days	500mg/L	91,4		[209]
	Copaifera epunctata Amshoff (Oleoresin)(referred to as *Copaifera reticulata*)	1h bath/day, for 3 days	100mg/L	48.5		[210]
	Piper hispidum Sw.	1h bath/d (3 baths -48h Interval between baths)	100mg/L	78.6		[212]
	Piper marginatum Jacq.	20min bath/d (3 baths -48h Interval between baths)	100mg/L	42,8		
Piscinoodinium pillulare(Schäperclaus) Lom 1981	***Piper aduncum*** L.	15min bath/day, for 3 days	20mg/L	83,8		[221]
Zeylanicobdella arugamensis Silva, 1963 (referred to as *Zeylanicobdella* sp.but according to WoRMS there is only one direct species within the genus)	***Curcuma longa***L.(referred to as *Curcuma domestica*)	30min	1.2ml/L	12.9	*Epinephelus fuscoguttatus x Epinephelus lanceolatus*	[222]
		60min	1.2ml/L	24		
	Salvia rosmarinusSpenn. (referred to as *Rosmarinus officinalis*)	60min	15ml/L	44.85		[223]

TableS5. Acute toxicity tests of different EOs and their major compounds for different fish species. Plant and Animal nomenclature in this work follows the Plants of the World Online (POWO) and World Register of Marine Species (WoRMS) databases (accessed on April 6th, 2025). If a species name cited in a reference, differs from, or is inconsistent with the updated POWO & WoRMS taxonomy, the referenced name is retained and noted accordingly. **LC_{50} (Lethal Concentration 50%)**: MAPs concentration in water that causes death in 50% of a test population within a specified period of exposure. It is commonly used in toxicology to assess the acute toxicity of chemicals, including pollutants, pesticides, or natural compounds such as EOs, especially in aquatic organisms like fish. The lower the LC_{50} value, the more toxic the substance is considered to be.

Fish Species	*Plant Nomenclature/* Active substance	Exposure Time	LC_{50}	Citation
Arapaima gigasSchinz, 1822	***Mentha × piperita***L.	4h	38.0mg/L	[228]
Betta splendensRegan, 1910	**Citrus × aurantium f. *aurantium***(referred to as *Citrus sinensis*)	48h	49.17 μL/L	[229]
	Syzygium aromaticum(L.) Merr. & L.M.Perry	48h	30.63mg/L	[230]
	Syzygium aromaticum(L.) Merr. & L.M.Perry (Eugenol)	48h	29.95mg/L	
Carassius auratus Linnaeus 1758	***Azadirachta indica***A.Juss (Azadirachtin)	3h	≈74mg/L	[177]
		4h	≈67mg/L	
		6h	≈60mg/L	
		12h	≈ 39mg/L	
		15h	≈22mg/L	
	Cinnamomum burmanni(Nees & T.Nees) Blume (referred to as *Cinnamomum cassia* -Cortex cinnamon (Cinnamaldehyde)	48h	13.34mg/L	[216]
		48h	59.66mg/L	
	Cinnamomum tamala (Buch.-Ham.) T.Nees & C.H.Eberm.	96h	15.05g/L	[231]
	Curcuma longa L. (Curcuma oil)	24h	31.73mg/L	[188]
		48h	28.85mg/L	
	Cymbopogon martini (Roxb.)Will Watson (Palmarosa oil) (referred to as *Cymbopogon martinii*)	24h	40.8mg/L	
		48h	39.15mg/L	
	Magnolia officinalis Rehder & E.H.Wilson (Mangolol)	96h	6.02mg/L	[193]
	Nigella sativa L. (Thymoquinone (1,4-benzoquinone))	96h	≈2.796 mg/L	[217]
	Swietenia mahagoni (L.) Jacq.	96h	11.39g/L	[231]
	Zanthoxylum asiaticum (L.) Appelhans, Groppo & J.Wen (Chelerythrine) (referred to as *Toddalia asiatica*)	48h	3.3mg/L	[197]
Channa punctataBloch, 1793	***Sapindus mukorossi*** Gaertn. (pericarp)	48h	3.5-10ppm	[232]
Clarias gariepinusBurchell 1822	***Aframomum melegueta*** K.Schum. (referred to as *Afranamum melegueta*)	96h	68.36mg/L	[186]
	Albizia gummifera (J.F.Gmel.) C.A.Sm.	96h	94.58mg/L	[233]
	Azadirachta indicaA.Juss	96h	319.38mg/L	[186]
	Melaleuca cajuputi Maton & Sm. Ex R.Powell	96h	127mg/L	[234]
	Phragmanthera capitata (Spreng.) Balle (referred to as *Phragmenthera capitata*)	96h	37.65mg/L	[235]
	Tephrosia vogelii Hook.f.	96h	277.82mg/L	[233]
Colossoma macropomumCuvier 1816	***Aloysia citrodora*** Paláu (referred to as *Aloysia triphylla*)	4h	109.57mg/L	[236]
	Lippia grata Schauer (referred to as *Lippia gracilis*)	4h	41.63mg/L	
	Piper aduncum L.	4h	48.17mg/L	
Ctenopharyngodon idellaValenciennes 1844	***Sophora flavescens*** Aiton(sophoraflavanone)	96h	46.6mg/L	[192]
Cyprinus carpioLinnaeus 1758	***Madhuca longifolia* var. *latifolia***(Roxb.) A.Chev. (referred to as *Madhuca latifolia*)	96h	26.79mg/L	[180]
	Syzygium aromaticum(L.) Merr. & L.M.Perry (referred to as *Eugenia aromatica*&*Eugenia caryophylata*)	96h	18.1mg/L	[237]
Danio rerioHamilton, 1822	***Cinnamomum verum***J. Presl (cinnamaldehyde)	96h	7.2ppm	[238]
	Eugenol	96h	21.0mg/L	[239]
	Melia azedarachL.	96h	51.41mg/L	[240]
	Syzygium aromaticum(L.) Merr. & L.M.Perry (referred to as *Eugenia aromatica*&*Eugenia caryophylata*)	96h	18.2mg/L	[241]
Danio rerioHamilton, 1822 (embryonic stages)	***Syzygium aromaticum***(L.) Merr. & L.M.Perry (referred to as *Eugenia aromatica*&*Eugenia caryophylata*)	144h	15.64mg/L	[242]
Danio rerioHamilton, 1822 .(Juveniles)		96h	18.8mg/L	

Epinephelus coioides Hamilton, 1822	***Allium sativum*** L.	96h	74.78mg/L	[243]
Epinephelus fuscoguttatus × Epinephelus lanceolatus	***Allium sativum*** L.	96h	993.11ppm	[244]
Gambusia affinis Baird & Girard, 1853	***Azadirachta indica*** A.Juss (Leaf Extracts)	24h	6.00ml/L	[245]
		48h	3.43ml/L	
		96h	3.00ml/L	
	Mallotus nudiflorus (L.) Kulju & Welzen (referred to as *Trewia nudiflora*)	96h	26537.05µg/L	[246]
	Zanthoxylum schreberi (J.F.Gmel.) Reynel ex C.Nelson (referred to as *Zanthoxylum monophyllum*)	240h (10d)	4234.07µg/ml	[247]
	Zanthoxylum schreberi (J.F.Gmel.) Reynel ex C.Nelson (referred to as *Zanthoxylum monophyllum*) (Germacrene D-4-ol)	240h (10d)	414.05µg/ml	
	Zanthoxylum schreberi (J.F.Gmel.) Reynel ex C.Nelson (referred to as *Zanthoxylum monophyllum*) (α-Cadinol)	240h (10d)	635.12µg/ml	
Heteropneustes fossilis Bloch, 1794	***Azadirachta indica*** A.Juss (Azadirachtin)	24h	173.06mg/L	[248]
		48h	80.69mg/L	
		72h	58.57mg/L	
		96h	52.35mg/L	
	Sapindus mukorossi Gaertn. (pericarp)	48h	3.5-10ppm	[232]
Ictalurus punctatus Rafinesque, 1818	***Rhus chinensis*** Mill. (pentagalloylglucose extracted from galls - referred to as *Gall chinensis*)	24h	151.3mg/L	[196]
Labeo rohita Hamilton, 1822	***Azadirachta indica*** A.Juss (Azadirachtin)	96h	44.61 ppm	[249]
Oncorhynchus kisutch Walbaum, 1792	Eugenol	96h	66.1mg/L	[250]
Oncorhynchus mykiss Walbaum 1792	***Carum carvi*** L.	96h	14.0mg/L	[251]
	Cuminum cyminum L.	96h	35.0mg/L	
	Thymus vulgaris L.	96h	6.6mg/L	
	Thymus vulgaris L. (Thymol)	96h	2.6mg/L	
Oreochromis niloticus Linnaeus 1758	***Achyranthes aspera*** L.	96h	1063.9mg/L	[252]
	Allium sativum L.	96h	0.4g/L	[184]
	Allium cepa L.	96h	3.54g/L	
	Azadirachta indica A.Juss (Leaf Extracts)	24h	6.4ml/L	[245]
		48h	3.22ml/L	
		48h	1.64 g/L	[253]
		96h	2.57ml/L	[245]
	Carica papaya L.	24h	1310.74mg/L	[254]
	Eugenol	10min	184.26mg/L	[255]
	Indigofera articulata Gouan(referred to as *Indigofera tinctoria*)	96h	1.3mg/L	[256]
	Ocimum gratissimum L.	96h	523.63mg/L	[257]
Piaractus mesopotamicus Holmberg, 1887	***Azadirachta indica*** A.Juss (Leaf Extracts)	96h	1.18- 1.20mg/L	[258]
Poecilia reticulata Peters, 1859	***Syzygium aromaticum*** (L.) Merr. & L.M.Perry (referred to as *Eugenia aromatica*&*Eugenia caryophylata*)	96h	21.7mg/L	[241]
Poecilia reticulata Peters, 1859 (females)	***Heterotheca inuloides*** Cass.	96h	103.67ppm	[259]
	Thymol	24h	12.51mg/L	[260]
	1,8-cineole	24h	3997.07mg/L	
Poecilia reticulata Peters, 1859 (fingerlings)	***Heterotheca inuloides*** Cass.	96h	12.39ppm	[259]
Poecilia reticulata Peters, 1859 (males)	***Heterotheca inuloides*** Cass.	96h	62.94ppm	
	Thymol	24h	10.99mg/L	[260]
	1,8-cineole	24h	1701.93mg/L	
Squaliobarbus curriculus Richardson, 1846	***Macleaya microcarpa*** (Maxim.) Fedde (dihydrosanguinarine)	48h	13.3mg/L	[191]
		48h	18.2mg/L	
Trichogaster fasciata Bloch & Schneider, 1801 (referred to as *Colisa fasciatus*)	***Euphorbia royleana*** Boiss.	24h	8.6ppm	[261]
	Euphorbia lactea Haw.	24h	7.6ppm	
	Euphorbia antisyphilitica Zucc. (referred to as *Euphorbia antisyphlitica*)	24h	16ppm	
	Jatropha gossypiifolia L. (referred to as *Jatropha gossypifolia*)	24h	37ppm	
